# Promoting healthy eating in early pregnancy in individuals at risk of gestational diabetes mellitus: does it improve glucose homeostasis? A study protocol for a randomized control trial

**DOI:** 10.3389/fnut.2023.1336509

**Published:** 2024-01-19

**Authors:** Emilie Bernier, Anne-Sophie Plante, Patricia Lemieux, Julie Robitaille, Simone Lemieux, Sophie Desroches, Ariane Bélanger-Gravel, Sarah Maheux-Lacroix, S. John Weisnagel, Suzanne Demers, Félix Camirand Lemyre, Mélanie Boulet, Jean-Patrice Baillargeon, Anne-Sophie Morisset

**Affiliations:** ^1^École de Nutrition, Université Laval, Québec, QC, Canada; ^2^Centre de Recherche du CHU de Québec-Université Laval, Québec, QC, Canada; ^3^Centre de Recherche Nutrition, Santé et Société (NUTRISS) de l’Institut sur la Nutrition et des Aliments Fonctionnels (INAF), Université Laval, Québec, QC, Canada; ^4^Faculté de Médecine, Université Laval, Québec, QC, Canada; ^5^Département de Communication, Université Laval, Québec, QC, Canada; ^6^Centre de Recherche de l’Institut Universitaire de Cardiologie de Pneumologie de Québec, Québec, QC, Canada; ^7^Département de Mathématiques, Université de Sherbrooke, Sherbrooke, QC, Canada; ^8^Centre de Recherche du CHU de Sherbrooke, Sherbrooke, QC, Canada; ^9^Centre Intégré Universitaire de Santé et de Service Sociaux de l'Estrie—CHU de Sherbrooke, Sherbrooke, QC, Canada; ^10^Département de Médecine, Université de Sherbrooke, Sherbrooke, QC, Canada

**Keywords:** healthy eating, diet quality, nutrition, pregnancy, gestational diabetes mellitus, randomized control trial, intervention

## Abstract

**Background:**

Healthy eating during pregnancy has favorable effects on glycemic control and is associated with a lower risk of gestational diabetes mellitus (GDM). According to Diabetes Canada, there is a need for an effective and acceptable intervention that could improve glucose homeostasis and support pregnant individuals at risk for GDM.

**Aims:**

This unicentric randomized controlled trial (RCT) aims to evaluate the effects of a nutritional intervention initiated early in pregnancy, on glucose homeostasis in 150 pregnant individuals at risk for GDM, compared to usual care.

**Methods:**

Population: 150 pregnant individuals ≥18 years old, at ≤14 weeks of pregnancy, and presenting ≥1 risk factor for GDM according to Diabetes Canada guidelines. Intervention: The nutritional intervention initiated in the first trimester is based on the health behavior change theory during pregnancy and on Canada’s Food Guide recommendations. It includes (1) four individual counseling sessions with a registered dietitian using motivational interviewing (12, 18, 24, and 30 weeks), with post-interview phone call follow-ups, aiming to develop and achieve S.M.A.R.T. nutritional objectives (specific, measurable, attainable, relevant, and time-bound); (2) 10 informative video clips on healthy eating during pregnancy developed by our team and based on national guidelines, and (3) a virtual support community via a Facebook group. Control: Usual prenatal care. Protocol: This RCT includes three on-site visits (10–14, 24–26, and 34–36 weeks) during which a 2-h oral glucose tolerance test is done and blood samples are taken. At each trimester and 3 months postpartum, participants complete web-based questionnaires, including three validated 24-h dietary recalls to assess their diet quality using the Healthy Eating Food Index 2019. Primary outcome: Difference in the change in fasting blood glucose (from the first to the third trimester) between groups. This study has been approved by the Ethics Committee of the Centre de recherche du CHU de Québec-Université Laval.

**Discussion:**

This RCT will determine whether a nutritional intervention initiated early in pregnancy can improve glucose homeostasis in individuals at risk for GDM and inform Canadian stakeholders on improving care trajectories and policies for pregnant individuals at risk for GDM.

**Clinical trial registration:**

https://clinicaltrials.gov/study/NCT05299502, NCT05299502

## 1 Introduction

### 1.1 Gestational diabetes mellitus prevalence, complications, and treatment

Gestational diabetes mellitus (GDM) is the most frequent health condition among pregnant individuals. Its prevalence is increasing worldwide, as it can reach up to 20% of pregnant individuals in some parts of the world, depending on their risk factors and the methods used to screen and diagnose GDM (1, 2). This condition is associated with adverse consequences such as fetal macrosomia, obesity, and glucose intolerance in the offspring, as well as higher risks of type 2 diabetes, metabolic syndrome, and cardiovascular diseases for the mother (3, 4). In addition, independently of a frank GDM diagnosis, even mildly elevated glucose levels can lead to negative effects on the health of the mother and child (5). Indeed, there is a strong and continuous association between third-trimester maternal glucose levels and increased birth weight as well as other pregnancy complications, even below the threshold of GDM diagnosis (5).

In Canada, routine screening for GDM is part of the usual pregnancy care and is recommended to all Canadian pregnant individuals between 24 and 28 weeks of gestation (6). The first-line treatment of GDM involves medical nutritional therapy and management provided by registered dietitians (RDs) in primary care, along with glucose self-monitoring (6). In practice, pregnant individuals with GDM are encouraged to follow the general recommendations of the 2019 Canada’s Food Guide (CFG), with particular attention to carbohydrate, fiber, and protein intake (6, 7). This type of intervention, alongside exercise, can be sufficient to improve the glycemic profile and to achieve glycemic goals in a high number of cases. However, for some individuals, pharmacological therapy is required to ensure the maintenance of adequate glycemic control (3, 6, 8). Moreover, nutritional therapy is generally initiated in the last trimester, thus limiting the potential benefits to a short period, and only those with a confirmed GDM diagnosis are referred to an RD. Therefore, most Canadian individuals at risk for GDM do not have access to nutritional therapy during their prenatal care. This situation is unfortunate because a high number of pregnant individuals, particularly those with a pre-pregnancy BMI ≥ 30 kg/m^2^, remain at an elevated risk for other pregnancy complications, including cesarean section births and higher birth weights (9).

Yet, the Diabetes Canada guidelines from 2018 are clear and state the need for an effective and acceptable intervention that will prevent the development of GDM, since such an approach has the potential to improve maternal and child health, with significant savings to the healthcare system (2). This raises the question of whether nutritional management in early pregnancy, initiated in at-risk individuals, could improve their glycemic profile.

### 1.2 Diet quality and glucose homeostasis during pregnancy

Recent evidence has demonstrated that healthy eating during pregnancy can have favorable effects on numerous maternal and neonatal outcomes during pregnancy (10). More importantly, better diet quality is shown to have positive effects on markers related to diabetes and is associated with lower GDM risk (11, 12). According to Gadgil et al. (13), even a modest improvement in diet quality could potentially contribute to achieving better glycemic control. Higher diet quality, characterized by a higher intake of plant-based and fiber-rich foods and a lower intake of red meats is associated with reduced GDM risk and is also in line with the 2019 CFG recommendations for better diet quality (11, 14, 15). Beyond the risk of GDM development during pregnancy, only a few studies have examined associations between diet quality and measurements of glucose homeostasis, such as fasting blood glucose and insulin levels in the gestational setting, and the results are inconsistent (16–18). For instance, a study by Martin et al. (17), which included more than 500 participants, indicated that the combined consumption of vegetables, fruits, whole grain foods, low-fat dairy products, breakfast bars, and water, was inversely associated with maternal insulin concentrations and insulin resistance. However, to date, no study has directly evaluated the effects of a nutritional intervention, aiming to improve diet quality, started in the first trimester on the glucose homeostasis of pregnant individuals at risk for GDM. Moreover, studies contributing to the literature regarding intervention to prevent women from developing GDM appear to have design limitations (19).

### 1.3 Nutrition in individuals at risk for gestational diabetes mellitus during pregnancy

Previous results from our team (20–22) and the results of other published studies (11, 12, 23–28) indicate that it is timely and innovative to conduct a trial aiming at improving diet quality and glycemic profile early in pregnancy. Namely, we observed a decline in diet quality during pregnancy, particularly among individuals with higher BMI and lower nutrition knowledge (20). Moreover, we noted an association between first-trimester diet quality and glucose homeostasis throughout pregnancy, indicating a better insulin sensitivity among French Canadians with a higher diet quality (22). These findings, along with the previous experiences of our team, provide a robust basis for the design and execution of this research.

A feasibility study from Finland evaluating the effects of a nutrition therapy to prevent inappropriate gestational weight gain (GWG) in pregnant individuals at high risk for GDM demonstrated that individualized dietary counseling provided by a clinical dietitian improved the quality of dietary fat intake (24). This shows that face-to-face interventions can realistically change eating behaviors and improve diet quality during pregnancy (24). However, this study did not provide any measurements of the glycemic profile of participants (24). A few years later, a study performed in China provided insights into the influence of a lifestyle interventions on GDM incidence and maternal outcomes, emphasizing the potential beneficial impact of dietary interventions during pregnancy (25). This lifestyle intervention, including individual education regarding balanced dietary patterns, the promotion of moderate physical activity, and weight control was associated with lower risks of GDM and other adverse maternal outcomes (25). Another study from China confirmed that diet and exercise interventions can reduce the incidence of GDM in pregnant individuals with high-risk factors by more than 25% (29). However, in both studies, adherence to healthy dietary patterns and physical activity were not assessed separately, thus limiting the possibility of disentangling the effects of diet and physical activity. Furthermore, the timing at which the intervention was started was not specified. Lastly, a protocol for a multicenter ongoing study in China was published last year (26). The authors propose to test individualized nutritional intervention started early in pregnancy in a high-risk population on GDM prevention (26). Other ongoing trials targeting GDM prevention in specific populations, such as South Asian pregnant individuals, further highlight the urgent need for reliable data and interventions (30).

Unpublished findings from a longitudinal study currently in progress in our team show higher diet quality scores during the first trimester compared to the preconception period. This is in line with findings stating that early lifestyle changes in pregnancy appear timely, as pregnant individuals expressed their desire and motivation to adopt healthier eating habits once pregnancy is confirmed (21, 31). Interestingly, a review published this year suggests that future trials targeting GDM should be initiated earlier, as early as 8–12 weeks of pregnancy (28). Another group recently demonstrated that immediate treatment of GDM before 20 weeks’ gestation led to a significantly, albeit modest, lower incidence of a composite of adverse neonatal events than those who received deferred or no treatment (32). The results of this trial support the observation that hyperglycemia often occurs before 24–28 weeks of gestation in individuals with GDM risk factors and confirm the need for further research regarding earlier management. Intervening early in pregnancy can allow for longer involvement and provide more counseling opportunities and activities. However, previous RCTs only dispensed intervention starting in the second trimester, which is unfortunate since shorter interventions limit the time in which improvements to modifiable risk factors can occur (28, 33). Even though some may argue that preconception care may be more beneficial in theory, it is not always realistic and achievable given the high prevalence of unplanned pregnancies, ranging from 25 to 40% in North America (34, 35). This leaves a missed window of opportunity to engage individuals in appropriate behaviors earlier in pregnancy during which they are particularly motivated to make changes (36). Starting interventions earlier can potentially lead to more significant improvements in modifiable risk factors for conditions like GDM (27). Therefore, RCTs studying interventions starting as early as possible, ideally in the first trimester, are needed and could provide critical insights into the effectiveness and safety of early interventions, helping to inform healthcare practices and policies for pregnant individuals and those planning to conceive.

There is thus a pressing need to gather reliable data about intervening early in pregnancy. Conducting such research within the Canadian population is crucial due to distinctive cultural and epidemiological characteristics, socio-economic factors, healthcare systems, and guidelines. For example, Canada is known for its cultural diversity, with a population comprising people from various ethnic backgrounds and cultural traditions. In contrast, some Asian countries may have more homogenous populations and cultural norms. Canada provides publicly funded healthcare services, including prenatal care, to all residents. In contrast, healthcare systems in some Asian countries may have different models of financing and service delivery. Genetic and environmental factors may contribute to variations in health conditions, such as GDM, and responses to nutritional interventions among different populations (37, 38). It is essential to recognize that dietary habits, culture, and healthcare practices significantly differ between Canadian and Asian populations, and these distinctions can have a substantial impact on the effectiveness and acceptability of nutritional interventions. Research tailored to the Canadian context can provide sensitive strategies that are more likely to be embraced and followed by the multicultural Canadian population. Results that are available, including those from our team, underscore the importance and potential of an intervention promoting healthy eating in early pregnancy to enhance the glycemic profile of individuals at risk for GDM.

### 1.4 Research questions and hypotheses

Given the significant impact of GDM and impaired glucose tolerance on both the mother and the child, it is necessary to develop and evaluate early nutrition interventions during pregnancy. In response to those considerations, we propose a nutritional intervention based on behavioral theory and the CFG, starting early in pregnancy and targeting glucose homeostasis. This paper describes the methodology of the *Saine Alimentation durant la GrossessE* (SAGE) trial.

We expect that early nutritional intervention (12–14 gestational weeks) will significantly improve: (1) changes in fasting plasma glucose between the first and third trimester, and (2) changes in 2-h postprandial glucose and other glucose homeostasis indices between the first and third trimester, compared to usual care. We also expect the intervention to improve changes in fasting glucose and other glucose homeostasis indices from the first to the second trimester (24–26 gestational weeks).

### 1.5 Study objective and outcomes

**Objective:** To examine whether an intervention promoting healthy eating initiated early in pregnancy improves glucose homeostasis in individuals at risk of GDM.

**Primary outcome:** Change in fasting plasma glucose (FPG) from the first to the third trimester.

**Secondary outcomes:**

- Changes in 2-h plasma glucose following the ingestion of 75 g of glucose from the first to the third trimester:

⚬ Glycemic response using the incremental area under the curve for glucose during the 2-h oral glucose tolerance test (OGTT).
⚬ Homeostatic Model Assessment for Insulin Sensitivity (HOMA-IS) index, which estimates the hepatic insulin sensitivity (39).
⚬ Matsuda index which estimates overall insulin sensitivity (hepatic and peripheral) (40).
⚬ Disposition index, which estimates beta-cell function, using Matsuda index*insulinogenic index (41).

- Changes in FPG and other indices listed above from the first to the second trimester.

**Exploratory outcomes:**

- Changes during pregnancy and from pregnancy to postpartum in physical activity, anxiety, and quality of life.
- Pregnancy and neonatal outcomes (i.e., GDM incidence, gestational weight gain, and birth weight).

## 2 Methods and analysis

### 2.1 Trial design and setting

This study is a unicentric, two-arm, parallel-group RCT comparing an intervention promoting healthy eating initiated early in pregnancy to usual care. The study will be conducted at the *Centre de recherche du CHU de Québec-Université Laval* in Quebec City, Quebec, Canada. In 2012, the year in which the most recent data is available, the prevalence of exposure to GDM in the Province of Quebec was 76 per 1,000 births (8%) (42).

### 2.2 Public and patient involvement

We have established partnerships with three patient partners, who are official collaborators of the study. They were all previously diagnosed with GDM and were trained by the Office of Patient Experience and Partnership Expertise. They have collaborated on this protocol on several levels: they provided input and feedback on the content of the nutritional intervention and video clips, on the acceptability of the different measures taken during the research project, and on the outcomes of the study. They have been and will be actively involved with our team throughout the study.

### 2.3 Participants eligibility

Individuals who meet the following inclusion criteria can participate in the study:

1. Aged ≥18 years;
2. Being at ≤14 weeks of gestation with a singleton pregnancy at the first visit (Visit 1 [V1]); and
3. Presenting a risk factor for GDM according to Diabetes Canada guidelines (2): being (a) 35 years of age or older or (b) from a high-risk group (African, Arab, Asian, Hispanic, Indigenous, or South Asian), or having (c) obesity (pre-pregnancy BMI ≥ 30 kg/m^2^), (d) prediabetes, (e) GDM in a previous pregnancy, (f) given birth to a baby that weighed more than 4 kg, (g) a parent, brother or sister with type 2 diabetes, (h) polycystic ovary syndrome, or (i) acanthosis nigricans.

Individuals presenting at least one of the following exclusion criteria will not be eligible to enroll in the study:

1. Having a diagnosis of overt diabetes mellitus or early GDM at V1.
2. Diagnosed with an active disease requiring nutritional therapy influencing glucose metabolism or with uncontrolled eating disorders that would interfere with the initiation of intervention.
3. Planning for or history of bariatric surgery, which would confound the impact of the nutritional intervention tested.
4. Planning for or engaging in another nutritional intervention program, which would also confound the impact of the nutritional intervention tested.
5. Using a medication affecting glucose metabolism (such as metformin or, corticosteroids), which may confound the primary outcomes of this study.
6. Inability to understand and communicate in French.
7. Unable to attend research visits at the participating center for the next 9 months.

### 2.4 Recruitment

Recruitment of eligible participants began in June 2022 and will be completed within 4 years. Participants are recruited from the greater Quebec City area and surrounding regions through targeted clinics or research centers, in collaboration with local physicians, nurses, and research investigators. Public printed material (posters displayed in hospitals, clinics, and community-based resources) and virtual advertising (email distribution or social media) are also used, as the latter seemed more efficient and effective than traditional offline methods among Canadian pregnant individuals (43). Thus, potentially eligible patients can be approached in one of two ways: (1) by a member within their circle of care who then provides contact info to research staff, or (2) by responding to an advertisement indicating their interest in learning more about the study. The initial screening for eligibility is conducted by research staff by phone via the administration of a questionnaire to confirm the inclusion and exclusion criteria. Written informed consent is obtained individually for each participant at the first visit (V1), after a full explanation of the study’s protocol by the research staff before any data collection or study procedures begin.

Final eligibility is confirmed by the site endocrinologist after V1 to ensure the absence of pre-existing diabetes mellitus or early GDM before randomization. Since there is no evidence to confirm whether the diagnostic criteria for tests performed between 24 and 28 weeks of gestation can be applied to tests performed in early pregnancy (32, 44), diabetes at this first visit will be diagnosed using the criteria for overt diabetes (diabetes present before pregnancy), as recommended by the latest Diabetes Canada guidelines (2). Hence, participants will be excluded from the project after their first visit if they have either FPG ≥ 7.0 mmol/L, HbA1c ≥ 6.5%, or 2-h plasma glucose following the ingestion of 75 g of glucose ≥11.1 mmol/L and will be referred and followed by a multidisciplinary team including RDs for prompt management (2, 45).

### 2.5 Participant timeline and data collection

**Figure 1:**
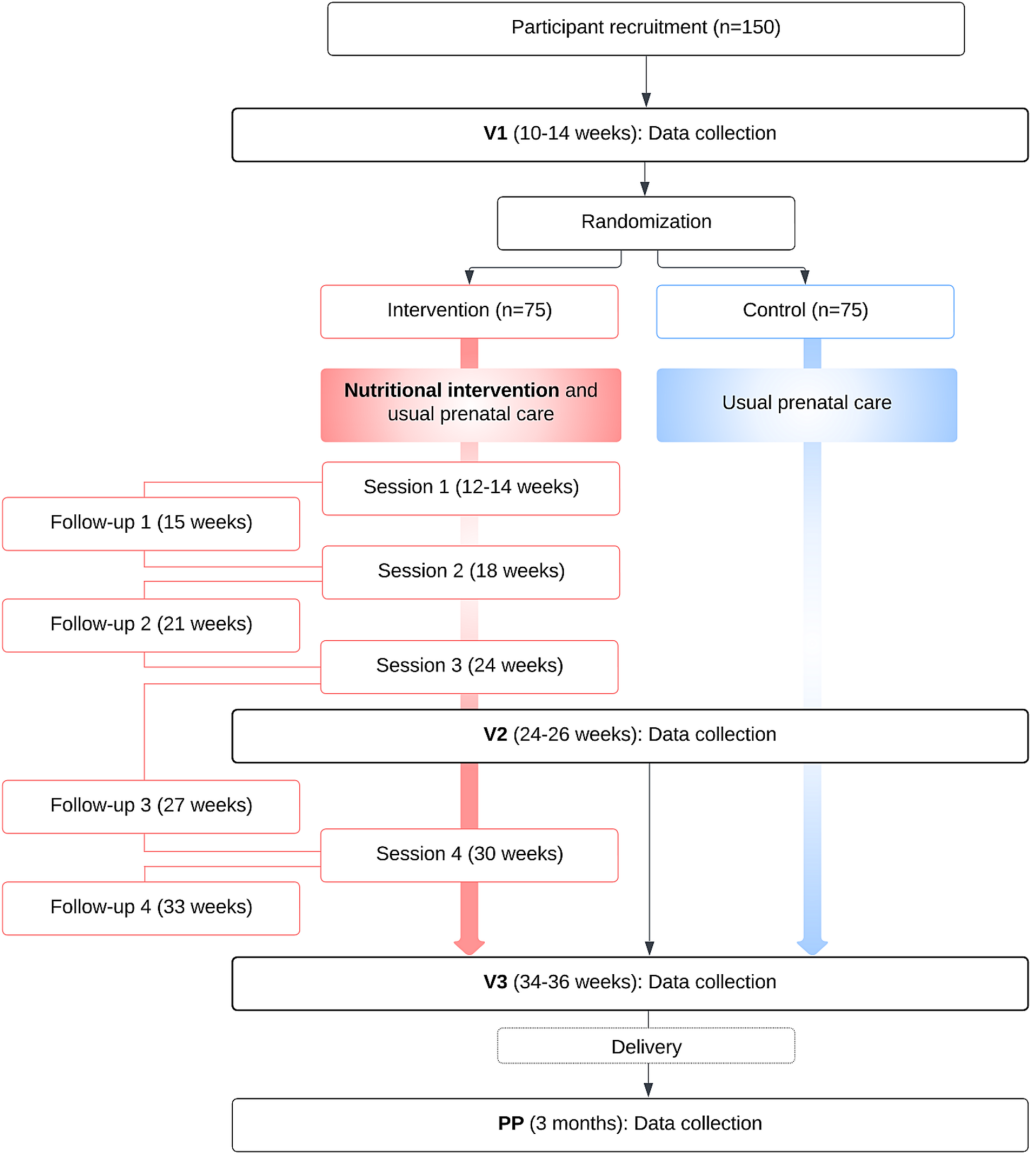
Participants’ timeline.

As illustrated in Figure 1, all participants will attend three on-site research visits (V1 at 10–14, Visit 2 [V2] at 24–26, and Visit 3 [V3] at 34–36 gestational weeks), during which a 2-h OGTT will be performed. At each trimester and 3 months after delivery, participants will also complete online questionnaires assessing various parameters (diet, dietary supplements and medication use, eating behaviors, physical activity, sociodemographic, medical history, anxiety during pregnancy, quality of life, motivation, social support, and satisfaction with the intervention). Data collection is outlined in Table 1 and methods of assessment are detailed in the following section. The total duration of participation is estimated at approximately 9 months, from the randomization at 12–14 gestational weeks to the last completion of questionnaires at 3 months postpartum.

**Table 1:** Data collection schedule.

Variable	Assessment method	Timing of data collection				
		V1	V2	V3	PP	End of the study
		10–14 weeks	24–26 weeks	34–36 weeks	12 weeks	
Informed consent	Information and consent form	⬤				
Sociodemographic status	In-house questionnaire	⬤				
Medical history	In-house questionnaire	⬤				
Medication use	In-house questionnaire	⬤	⬤	⬤		
Anthropometric outcomes						
Height	Stadiometer	⬤				
Weight before pregnancy	In-house questionnaire	⬤				
Weight	Scale	⬤	⬤	⬤		
Glucose homeostasis outcomes						
Glucose^*^	Blood sample	⬤	⬤	⬤		
Insulin^*^	Blood sample	⬤	⬤	⬤		
C-peptide^*^	Blood sample	⬤	⬤	⬤		
HbA1c	Blood sample	⬤				
Lifestyle outcomes						
Dietary intakes	R24W	⬤	⬤	⬤	⬤	
Diet quality	HEFI-2019	⬤	⬤	⬤	⬤	
Dietary supplements use	In-house questionnaire	⬤	⬤	⬤	⬤	
Eating behaviors	DEBQ	⬤	⬤	⬤	⬤	
Intuitive eating	IES-2	⬤	⬤	⬤	⬤	
Physical activity	PPAQ/IPAQ	⬤	⬤	⬤	⬤	
Perceived stress	PSS-10	⬤	⬤	⬤	⬤	
Anxiety during pregnancy	PRAQ-R2	⬤				
Quality of life	SF-36	⬤		⬤		
Motivation	REBS	⬤		⬤		
Social support	In-house questionnaire			⬤		
Satisfaction with the intervention^†^	In-house questionnaire			⬤		
Pregnancy outcomes						
Obstetrical history	In-house questionnaire	⬤				
Nausea and vomiting	In-house questionnaire	⬤	⬤	⬤		
Craving and aversions	In-house questionnaire	⬤	⬤	⬤		
Gestational weight gain	Patient medical record					⬤
Pregnancy complications	Patient medical record					⬤
Gestational age at delivery	Patient medical record					⬤
Delivery mode	Patient medical record					⬤
Birth outcomes	Patient medical record					⬤

^*^Collected after a 12-h fast, as well as 15, 30, 60, and 90, 120 min after the ingestion of 75 g of glucose. ^†^Evaluated for the participants receiving the intervention only. DEBQ, Dutch Eating Behaviors Questionnaire; HbA1c, Glycated hemoglobin; HEFI-2019, Healthy Eating Food Index 2019; IES-2, Intuitive Eating Scale; IPAQ, International Physical Activity Questionnaire; PP, postpartum virtual data collection; PSS, Perceived Stress Scale; PPAQ, Pregnancy Physical Activity Questionnaire; PRAQ-R2, Pregnancy Related Anxiety Questionnaire; R24W, Web-based 24-h dietary recalls; REBS, Regulation of Eating Behavior Scale; SF-36, 36-Item Short Form Survey; V1, V2, and V3, research visits at the first, second and third trimester.

### 2.6 Randomization

Randomization occurs after the eligibility assessment and completion of the V1 and associated questionnaires mentioned below. Group allocation is concealed using online computerized randomization by FANI (*Application Fonctionnelle de Nutrition sur Internet*), a Web platform developed and hosted by the Institut sur la nutrition et les aliments fonctionnels, Université Laval. Eligible subjects will be randomized to the intervention or control group at a ratio of 1:1. The randomization order is generated independently using online computerized group allocation with a permuted block of variable block sizes (4–10). The participant’s treatment allocation is then automatically and electronically delivered to the local site investigator and research staff. Randomization is performed over the phone with the candidate after confirmation of her willingness to participate. Group allocation is then provided immediately to the participant.

#### 2.6.1 Intervention arm

The development of the suggested intervention was intricately woven with principles drawn from a conceptual model based on health behavior change theory during pregnancy and illustrates some key elements with motivation and self-efficacy as central health behavior change constructs (46) in facilitating the transformation of pregnant individuals’ dietary habits. Furthermore, the intervention was thoughtfully aligned with the latest guidance provided by the CFG. It encompasses a three-tiered approach designed to empower expectant mothers with the knowledge, skills, and motivation necessary for optimal diet during pregnancy.

The main component of the suggested intervention is four individual 1-h in-person (or virtual, according to participants’ preference) counseling sessions conducted by an RD at key junctures in the pregnancy journey (12, 18, 24, and 30 weeks). The RD uses motivational interviewing which has been has been shown to have had promising results when used in nutritional interventions (47–50). Nutritional counseling during pregnancy is not only the most effective intervention for improving pregnant women’s knowledge and understanding but could also prevent multiple perinatal complications (51–53). Within the individualized counseling sessions, participants will be encouraged to elaborate Specific, Measurable, Attainable, Relevant, Timely (S.M.A.R.T.) goals regarding their eating habits per the CFG guidelines. Hence, every participant’s goals will be tailored to their own verbalized priorities and motivations to improve compliance. During these counseling sessions, the RD will also help participants to identify potential barriers and solutions to achieve their goals. Between the individual counseling sessions, four phone call follow-ups with the RD will be provided to review the goals and solutions chosen by participants (15, 21, 27, and 33 gestational weeks).

The intervention also includes access to 10 informative brief video clips about healthy eating during pregnancy, which are hosted on a private Web platform. The video clips were developed by a team of RDs from our research team, in line with the data demonstrating that (1) education is a necessary component for behavior change (54), and (2) the lack of available resources appears to be a key barrier to healthy eating (55, 56). Their conception also involved experts in prenatal nutrition, communication, and three patient partners. The content was based on current evidence, as well as, on national and provincial recommendations including the CFG and both Health Canada and Quebec National Institute of Public Health’s guidelines for pregnancy. These brief (4–14 min) and informative nutritional video clips cover concerns about healthy eating throughout pregnancy and aim to increase nutrition knowledge to improve motivation (57) and facilitate the achievement of their set goals. The video clips have the following title and the development process as well as their content is available in Supplementary Tables S1, S2.

1. The importance of healthy eating during pregnancy
2. Should we eat differently during pregnancy?
3. General recommendations for healthy eating
4. Food precautions to take during pregnancy
5. Healthy eating habits
6. Why and how to favor minimally processed foods?
7. The influence of the food environment
8. Discomforts during pregnancy: what is the role of food?
9. Nutrients for a healthy diet
10. A better understanding of nutrition labeling

Lastly, participants randomized to the intervention group are invited to join an online private virtual community on Facebook to discuss and share experiences, thus maintaining motivation and providing social support, as requested by pregnant individuals (58). The Facebook group is animated and moderated by an RD. Each week, the RD shares a new publication that addresses one of the four dimensions of social support (emotional, instrumental, informative, and evaluative) (59) to guide the discussion between participants. This virtual support community was established to provide continuous motivation, peer support, and the exchange of experiences to bolster participants’ commitment to their nutritional goals.

This multi-dimensional approach aims to empower pregnant individuals to make positive and sustainable dietary choices throughout their pregnancy. Moreover, this intervention has been strategically designed to encompass a multitude of behavior change techniques, including goal setting, education, problem-solving, coping planning, and social support (60). By incorporating these techniques, the intervention aims to create a comprehensive framework that not only educates and informs but also enables and supports pregnant individuals in their journey toward healthy eating during pregnancy. These techniques are thoughtfully intertwined within the counseling sessions, the video clips, and the virtual support community, fostering motivation, self-efficacy, and the development of practical skills necessary for achieving and maintaining optimal dietary practices. The synergistic effect of these techniques provides a robust foundation for behavior change, ultimately enabling participants to make informed choices and establish healthy eating habits during pregnancy.

The chosen time frame for intervention commencement (12 weeks), was determined in accordance with the Canadian perinatal care practices. In most cases, contact with medical professionals for pregnant individuals does not occur until around 10–12 weeks, coinciding with the confirmation of pregnancy through ultrasound assessments, such as nuchal translucency and dating ultrasound. Additionally, it is well-acknowledged in medical literature that a significant proportion of miscarriages occurs within the first 12 weeks of pregnancy. Considering these factors, initiating the intervention at 12 weeks allows for a more stable participation, reducing the risk of early dropouts due to miscarriage.

The chosen hybrid mode of delivery, combining in-person, virtual, and phone counseling, is based on previous studies suggesting that in-person counseling with multiple visits might not be feasible and can decrease treatment efficacy (61). The use of Web-based resources to provide nutrition information is based on the reported preferences of French-Canadian pregnant individuals (62). Data also suggest that evidence-based online educational interventions are credible, low cost, and easily accessible, while also complementing the advice provided by healthcare providers (56).

Our choice to prioritize nutrition intervention, rather than covering areas such as physical activity or other lifestyle habits, is primarily based on the simplification of the intervention that takes into account the reality that pregnant individuals are often faced with a multitude of responsibilities and concerns, which can lead them to feel overwhelmed, especially as their time to meet with several health professionals is limited (63). This simpler approach aims to enhance adherence to the program and facilitate the achievement of individual goals and have the potential to be implemented with perinatal care. This choice is also based on the fact that diet-based interventions have been associated with a reduced risk of GDM while mixed-approach interventions seem to be less effective (64). Moreover, current literature clearly demonstrates that pregnant individuals are motivated to eat well during pregnancy and require help to do so (65, 66). Finally, by choosing to target nutrition, we also avoid having to interrupt the intervention due to physical activity contraindications (67, 68). However, we do not deny the importance of staying active during pregnancy, which is suggested by recent Canadian recommendations (67). Physical activity will be measured every trimester to control for this confounding factor. Participants having questions about physical activity will be referred to their general practitioner or a kinesiologist.

#### 2.6.2 Control arm

Participants randomized to the control group receive usual prenatal care from their general practitioner without access to the nutritional intervention. The usual prenatal care provided to all pregnant individuals in the Province of Quebec is provided by general practitioners, midwives, and obstetricians in private practice, family medicine groups, or in hospitals or birthing centers. Pregnant individuals in Quebec are encouraged to seek prenatal care early in their pregnancy and to meet with a healthcare provider or midwife as soon as the pregnancy is confirmed. Individuals have the option to choose between receiving care from a family doctor, obstetrician, or midwife, depending on their preferences and specific health needs. Midwives provide care as part of a team approach and are available for low-risk pregnancies. Throughout pregnancy, individuals attend a series of prenatal visits with their chosen healthcare provider. The frequency of these visits may vary but typically consists of monthly visits in early- and mid-pregnancy and bimonthly visits in late pregnancy. These visits generally include health assessments, monitoring of fetal development, discussions about pregnancy-related topics, and various routine tests and screening, such as ultrasound and blood tests. Usual prenatal care also involves education and counseling on topics such as childbirth preparation, breastfeeding, and postpartum care. Expectant mothers can also receive guidance through web-based or printed documents from national, provincial, or local resources, which can provide information on the reasons and ways to achieve and maintain a balanced diet and proper nutrition during pregnancy. They may be referred to RDs if considered necessary by their healthcare provider. In general, individuals carrying more than one fetus and those with complex medical histories (including a history of bariatric surgery or eating disorders), pre-existing health problems and GDM can be referred to an RD during pregnancy for appropriate dietary management. As a result, only a small proportion of pregnant individuals in Quebec have access to nutritional management as part of their usual prenatal care.

#### 2.6.3 Both groups

Participants with a positive diabetes diagnosis in the first trimester are excluded. However, if a GDM is diagnosed during the second or third research visits, participants remain in the project. In the second and third trimesters (V2 and V3), GDM will be diagnosed using the alternate one-step approach suggested by Diabetes Canada’s latest guidelines and the International Association of the Diabetes and Pregnancy Study Groups based on the Hyperglycemia and Adverse Pregnancy Outcome study (5). Therefore, GDM will be diagnosed if one plasma glucose value is abnormal (FPG ≥ 5.1 mmol/L, 1 h following the ingestion of 75 g of glucose ≥10.0 mmol/L, 2 h following the ingestion of 75 g of glucose ≥8.5 mmol/L) (2). The usual conduct for GDM management is to refer to the multidisciplinary team for pregnancies at risk in hospitals or clinics and nutritional therapy is usually initiated within a few weeks (2). Hence, participants diagnosed with GDM will be referred and followed by a multidisciplinary team including RDs, regardless of their group allocation. Since the diagnosis of GDM in the second trimester and the resulting treatment received by the participants may interfere with our results, the effect of this added care will be monitored through retrospective patient medical record data collection and considered in our statistical analyses. However, a late diagnosis at V3 and its subsequent management are not expected to interfere with the results collected at V3 and will therefore not be considered in analyzing our primary and secondary outcomes. Participants are also instructed to contact the research staff between visits or phone calls if they are diagnosed with GDM outside of research visits or if any relevant situations occur during their participation, making them no longer eligible for the study or unable to complete their participation.

### 2.7 Study outcomes assessments

#### 2.7.1 Glucose homeostasis outcomes

At each visit, glucose, insulin, and C-peptide will be measured on fresh blood samples drawn after a 12-h fast, as well as 15, 30, 60, and 90, 120 min after the ingestion of 75 g of glucose. Glucose will be measured enzymatically by the hexokinase method whereas insulin and C-peptide will be measured with an electrochemiluminescence immunoassay. The obtained data will be used to calculate the incremental area under the curve for glucose (iAUC glucose) using the trapezoid method from 0 to 120 min. The following indexes will also be calculated: HOMA-IS [Fasting insulin (μU/ml) × fasting glucose (mmol/L)/22.5] (39); Matsuda index {10,000/[fasting glucose × fasting insulin × (mean glucose × mean insulin)]^1/2^} (40); Insulinogenic index [(insulin at 30 min − fasting insulin)/(glucose at 30 min fasting glucose)] (41). The disposition index, which serves as an integrated measure that reflects beta-cell function, will be calculated using Matsuda index × insulinogenic index (41). GDM incidence will be recorded throughout the study based on OGTTs performed during the research visits or based on medical records if not diagnosed during research visits detailed (2).

#### 2.7.2 Dietary outcomes

Three automated, self-administered, and validated 24-h dietary recalls (R24W) using the *Rappel de 24 heures Web* virtual platform, developed and hosted by the *Institut sur la nutrition et les aliments fonctionnels*, at *Université Laval* and validated among Canadian pregnant individuals (69–71). It will be used to measure food intake at each trimester and 3 months postpartum. The R24W platform allows the research staff to randomly select 3 days (2 weekdays and 1 weekend day) in each trimester. On the days the R24W needs to be filled, an email will be sent to the participants, asking them to record all foods and beverages they had the day before (24-h period). Individuals who do not have access to a computer, tablet, or cell phone to complete these online questionnaires (and other questionnaires detailed below) will be invited to complete them on-site, during their on-site visit or the following days at the research center, where a tablet will be provided for the time needed to complete the questionnaires. The 2018 version of the 2015 edition of the Canadian Nutrient File (72) is used to code every food item that is offered on the web platform, enabling the automatic extraction of nutritional data. If a participant fails to complete the R24W, subsequent emails will be sent, within the trimester, until three R24Ws are completed.

Diet quality will be measured using the Healthy Eating Food Index 2019 (HEFI-2019) score, using the mean intakes from repeated R24W (73). This score was recently developed and validated among Canadian adults (74). The HEFI-2019 includes 10 components, of which five are based on foods, one on beverages, and four on nutrients, with a total score of 80 points, with a higher score representing a greater level of adherence to the CFG recommendations on healthy food choices. Since the intervention is based on the 2019 Food Guide, we believe this is the best index to use.

#### 2.7.3 Quality of life

Quality of life will be evaluated in the third trimester using the 36-Item Short Survey (SF-36) (75), which has been previously used among pregnant populations across the globe (76, 77). The SF-36 includes a multi-item scale that assesses eight health concepts: (1) limitations in physical activities because of health problems; (2) limitations in social activities because of physical or emotional problems; (3) limitations in usual role activities because of physical health problems; (4) body pain; (5) general mental health (psychological distress and well-being); (6) limitations in usual role activities because of emotional problems; (7) vitality (energy and fatigue); and (8) general health perceptions.

#### 2.7.4 Acceptability and feasibility of the intervention

In this prospective study, we will thoroughly assess both the acceptability and feasibility of the proposed nutritional intervention. To evaluate acceptability, we will employ a mixed-methods approach. Quantitative data will be collected through a 38-item in-house questionnaire among participants receiving the intervention only. This survey aims to capture their perceptions of the intervention’s relevance and appropriateness, and to gather information on their overall satisfaction with the components of the intervention, including the individual counseling sessions, the video clips, and the virtual community, using Likert scales. A content analysis of the Facebook group could also provide qualitative insights into participant engagement and interactions within the community, assessing the depth of discussions, sharing of experiences, and support provided. Attendance to the sessions with the nutritionist will be recorded, as well as their duration. Participants will be questioned regarding their viewing of the video clips at each research visit and intervention follow-up. The engagement metrics on the virtual community include observations such as participation (i.e., if the participant have joined the Facebook group or not), as well as, the number of views, likes, and comments by participants. This multi-faceted approach provides a comprehensive view of participant adherence and engagement with each intervention component throughout the study. Feasibility will be assessed by comparing recruitment rates to predetermined targets. Reasons for non-participation will also be collected from eligible participants who declined participation. Additionally, participant retention rates will be monitored throughout the study, considering a dropout rate of less than 20% as indicative of feasibility. Barriers to participation and reasons for drop-out will also be recorded. Adherence to the intervention will be assessed using activity logs and tracking of engagement metrics within the platform on which the video clips are hosted and the virtual community. An assessment of the intervention’s compatibility with the protocol, available resources, and timeline will be conducted through continuous process evaluations. Therefore, we plan to document resource utilization (time, personnel, and financial resources required for intervention delivery) to compare these to the initially projected allocation and to assess whether the intervention was delivered within the planned time frame, identifying any delays or deviations from the study schedule. This will allow us to comprehensively assess intervention delivery, identifying challenges, successes, and opportunities for refinement with the research team and setting. The outcomes of these evaluations will be pivotal in refining the RCT design, optimizing intervention delivery, and enhancing overall study viability throughout the project.

#### 2.7.5 Other variables

##### 2.7.5.1 Sociodemographic status

A socio-demographic web questionnaire including questions on age, ethnicity, education, employment status, relationship status, parental status, family income, and place of residence will be completed. Recognizing that individuals who do not identify as female also have perinatal needs and experiences that may be similar to, but also different from cisgender women (78), an additional question was added to collect information on participants’ gender identity.

##### 2.7.5.2 Medical history, medication, and dietary supplements used

A self-administered questionnaire will be used to evaluate the participants’ relevant medical history, use of medication, natural products, and dietary supplements.

##### 2.7.5.3 Anthropometric measurements

Height and pre-pregnancy weight will be self-reported by participants at screening. Height will be confirmed at V1 by a trained research assistant using a stadiometer. Weight is measured at each research visit using a scale. Pre-pregnancy BMI [weight (kg)/height (m)^2^] will be calculated using self-reported pre-pregnancy weight and on-site measured height. GWG will be calculated using body weight values during pregnancy collected retrospectively from medical records. Total GWG will be calculated as the difference between the last measured body weight value and the self-reported pre-pregnancy weight.

##### 2.7.5.4 Eating behaviors and Intuitive eating

The Dutch Eating Behaviors Questionnaire (DEBQ) will be used to measure participants’ eating behavior at every trimester and 3 months postpartum (79). This self-administered 33-item tool assesses three distinct eating behaviors in adults: (1) emotional eating; (2) external eating; and (3) restrained eating. Intuitive eating will be assessed at every trimester and 3 months postpartum using the Intuitive Eating Scale-2 (IES-2) (80, 81), used and validated in pregnancy (82). This 23-item questionnaire evaluates four subscale scores as well as a total intuitive eating score summing the four subscale scores: (1) eating for physical rather than emotional reasons; (2) unconditional permission to eat; (3) reliance on internal hunger and satiety cues; and (4) body−food choice congruence.

##### 2.7.5.5 Physical activity

Physical activity behavior during every trimester will be assessed online using a self-administered French translated and validated version of the Pregnancy Physical Activity Questionnaires (PPAQ) (83, 84). The PPAQ version used in this study consists of a 33-item semiquantitative questionnaire that asks respondents to report the time spent participating in activities including household/caregiving, occupational, sports/exercise, transportation, and inactivity. At 3 months postpartum, the physical activity habits during the last 7 days will be assessed using the International Physical Activity Questionnaire (IPAQ) (85).

##### 2.7.5.6 Perceived stress and anxiety during pregnancy

The Perceived Stress Scale (PSS-10) will be used to assess stress levels at every trimester and 3 months postpartum, which has been widely used in both the prenatal and postpartum periods (86). The Pregnancy-Related Anxiety Questionnaire (PRAQ-R2) will be used to specifically evaluate participants’ anxiety in their first trimester (87). This 10-item tool assesses three dimensions of pregnancy-related anxiety: (1) fears about fetal health; (2) fear of childbirth; and (3) concerns about their physical appearance.

##### 2.7.5.7 Motivation

Since intrinsic motivation is a key concept of the theoretical model we use and is used as a lever to achieve the objectives of the intervention (46), it will be measured in the first and the third trimester using the Regulation of Eating Behavior Scale (REBS) (88). This scale evaluates the different behavioral regulatory styles: (1) intrinsic motivation; (2) the four types of extrinsic motivation (integrated, identified, introjected, and external regulation); and (3) motivation.

##### 2.7.5.8 Social support

A self-administered two-item questionnaire will be used to measure the perceived social support received by participants on improving their eating habits in the last 6 months. The questions in this tool are designed to assess: (1) the sources of social support participants could count on to help them improve their eating habits as well as (2) their level of satisfaction with the support received.

##### 2.7.5.9 Pregnancy outcomes

A self-administered questionnaire will be used in the first trimester to evaluate the participants’ obstetrical history. At every trimester, participants will also complete a questionnaire collecting information about frequent prenatal discomforts that could impact their dietary habits, such as nausea, and vomiting, as well as cravings and aversions. Other variables will also be collected in medical records at the end of the study, pregnancy complications and management, gestational age at delivery, delivery method, as well as birth outcomes (i.e., neonatal anthropometric measures, delivery complications).

##### 2.7.5.10 Variables collected during follow-ups

Variables collected during the nutrition counseling sessions and phone follow-ups, such as the duration of the meeting, the type of goals (i.e., specific food groups, purchase of certain foods or frequency of restaurants, etc.), and the level of goal achievement will also be collected retrospectively from the participant research record.

### 2.8 Data management and monitoring

The research measures and outcomes are recorded using online questionnaires and paper forms at specific time points. Research team members ensure the integrity of the data before entering it into the FANI centralized web-based database. The research coordinator is responsible for training research staff and health professionals (dietitians and nurses) involved in the research project, as well as monitoring activities.

The trial steering committee includes the principal investigator, two co-investigators with complementary expertise in GDM management, two external investigators with extensive experience in clinical trial methodology, a dietitian involved in health service delivery, an engaged patient-partner, the study coordinator and research team members. Their involvement began during the protocol design stage, and they meet periodically to provide guidance and to support the coordination of the study. Formal meetings will be held twice a year to review the progress of the study and any problems that may arise in the conduct of the study. The following potential issues will be discussed: recruitment, ineligibility, protocol violation, adverse effects, dropout rates, completeness and timeliness of data, and balance between study arms. The coordinator will be responsible for scheduling the trial steering committee meetings.

The data monitoring committee will include the principal investigator, two external physicians and clinical investigators with extensive experience in diabetes and pregnancy follow-ups, and the study coordinator. Formal meetings will be held to review any clinical problems that arise in the conduct of the study if needed.

#### 2.8.1 Participants’ safety and adverse effects monitoring management

Even though the proposed project is not expected to pose any risks for participants, the proposed research protocol incorporates measures to ensure participant safety and minimize risks associated with the nutritional intervention and other study procedures. Trimestrial administration of standard 2-h OGTT is considered safe for both the expecting mothers and their fetuses, as this test is performed as part of usual prenatal care. Data monitoring will be constantly done by the treating physicians and the results of all OGTTs will be validated by an endocrinologist. Hence, participation in this project can allow early identification and prompt management of GDM in some participants. The nutritional intervention provided aims to improve diet quality and glycemic profile. Thus, it is very unlikely to cause any safety issues as it is already recommended during pregnancy in individuals with GDM. Moreover, since this intervention does not involve any caloric restriction or strict recommendations, it is not expected to pose any risks such as insufficient weight gain or ketonuria for the participants.

Since the study is open-label, even if very unlikely, it will be easy to determine whether clinical problems that occur are related to the intervention. However, to ensure safety monitoring, adverse events will be closely monitored. Adverse events will be classified into two categories:

- Adverse Events of Special Interest refer to any undesirable occurrence experienced by a study participant during the trial, whether or not considered related to the provided intervention, and include the following:

⚬ vomiting during the oral glucose tolerance test;
⚬ loss of consciousness during the oral glucose tolerance test;
⚬ thromboembolic event during pregnancy;
GDM;
⚬ gestational hypertensive disorder (gestational hypertension, preeclampsia, and eclampsia);
⚬ preterm birth (occurring after 22 weeks and before 37 weeks of gestational age); and
⚬ spontaneous abortion or miscarriage (i.e., < 22 weeks of gestational age).

- Serious Adverse Events are defined as any untoward medical occurrence that results in inpatient hospitalization or prolongation of existing hospitalization, severe congenital malformation or death, whether or not considered related to the provided intervention, and include the following:

⚬ hospitalization (other than for childbirth);
⚬ late fetal loss (i.e., fetal death between 22 and 28 weeks of gestational age);
⚬ stillbirth (i.e., > 28 weeks of gestational age);
⚬ neonatal death (between birth and before 28 days of life);
⚬ newborn with severe congenital malformation (causing functional disability); and
⚬ admission of the newborn to the intensive care unit.

During their participation, all study participants will be closely monitored for the occurrence of adverse events. Upon identification of an adverse event, the investigator will assess its severity (mild, moderate, or severe), its expectancy, and its causality. If needed, the investigator will take appropriate actions. The trial steering and monitoring committees will also be notified with relevant information on adverse events for their review.

### 2.9 Bias and retention strategies

Due to the nature of the study, blinding of the research team and participants will not be possible. However, the online computerized allocation used will prevent allocation bias. Ascertainment bias cannot be prevented as this RCT involves a treatment in which active participation is necessary and participants cannot be blinded to the intervention or the GDM diagnosis. Withdrawal bias will be assessed by appropriate data analyses. Efforts will be made to eliminate publication bias through the registration of our trial at inception (NCT05299502) and publication of the results in peer-reviewed journals. Finally, outcomes are objective and standardized, and hence difficult to bias.

The expected rate of withdrawal and loss to follow-up is approximated at 10% based on similar trials during pregnancy (24, 26, 89). A greater loss to follow-up is expected in the control group, as observed in other RCTs (24, 26, 89). Conservatively, we calculated a sample reduction of 20%.

To maximize retention, for both groups, we will ensure a close and personalized follow-up for each participant and all questions and concerns will be answered promptly. A financial compensation of $20 is also offered to participants to thank them for attending each research visit. For the control group, they are offered a free 1-h consultation with an RD from our team, upon completion of the study. For the intervention group, the fact that the RD is always available via the Facebook community will be an advantage for the retention of our participants. The option to meet with the RD virtually also represents a benefit. As previously mentioned, nutritional intervention compliance, as well as attendance at each consultation will be recorded. Video clip views and engagement to the Facebook group will also be tabulated (likes, comments, and views). Based on RCTs during pregnancy (90) and our previous studies, we conservatively expect a compliance rate of 85%.

### 2.10 Sample size calculation and statistical analysis

#### 2.10.1 Sample size calculation

The sample size calculation for our study is predicated on relevant prior research (5, 91). First, data from one of our previous studies conducted among a sample including pregnant people at risk for GDM indicates that the mean change in FPG from the first to the third trimester was an increase of 0.4 ± 0.8 mmol/L, which is what we expect to find in the control group (91). Second, it was previously demonstrated that an increase of 0.4 mmol/L (1 SD) in FPG in the third trimester is significantly associated with higher odds of unfavorable perinatal outcomes. Our sample size calculation was therefore primarily centered on these findings, as it aims to enable the detection of a minimal clinically significant difference between groups, specifically a 0.4 mmol/L change in delta FPG levels from the first to the third trimester. We postulate that the control group will experience an FPG increase of 0.4 ± 0.8 mmol/L, per previous observations, while the intervention group is anticipated to maintain stable FPG levels (0.0 ± 0.8 mmol/L) (effect size of 0.5). To ensure a statistical power of 80% in detecting this clinically significant 0.4 mmol/L difference between groups, with a significance level (alpha) set at 0.05, we have factored in two considerations. First, we assume a 4% pregnancy loss (92), and an 8% premature delivery (93). Second, given the nature of the study and drawing from experiences and other relevant research, we conservatively anticipate a dropout rate of 20% (24, 26, 89). Consequently, we are in the process of recruiting a total of 150 pregnant women for our study.

#### 2.10.2 Statistical analyses

The primary analyses will follow the complete case intention-to-treat principle. Changes from first- to third-trimester measurements of the primary and secondary outcomes between the intervention and control groups will be assessed using a two-sample one-sided *t*-test for means. To determine whether the results obtained depend on an improvement in diet quality, a generalized linear model will be used. Sensitivity analyses will be performed on all outcomes to (1) determine if missingness of the outcome data is associated with prognostic characteristics at baseline and treatment group; (2) compare the results obtained with those following an intention-to-treat analysis where missing outcomes are handled using multiple imputation by chained equations, an analysis on a complete case per protocol, and a per-protocol analysis where missing outcomes are handled using multiple imputation by chained equations; and (3) to explore potential sources of heterogeneity in treatment effects. We thus plan to test the effect of various covariables including age, pre-pregnancy BMI, parity, gestational weight gain, GDM risk factors, fasting blood glucose in the first trimester, GDM diagnosis in the second trimester, adherence to the intervention (with a weighted model based on the level of participation in the intervention), and intervention delivery mode (in person of virtually). Those analyses will ensure a more comprehensive understanding of how different factors may influence the intervention’s outcomes in diverse subgroups of the study population. Programming codes will be tested with a dummy data set to ensure their validity. No preliminary or interim analyses are planned. An exploratory health-economic analysis could be conducted *post hoc*.

## 3 Discussion

This paper presents the research protocol for a unicentric two-arm parallel-group RCT evaluating the effects of an intervention promoting healthy eating initiated in early pregnancy on the glucose homeostasis of individuals considered at risk of GDM (*Saine Alimentation durant la GrossessE*). The nutrition intervention is based on CFG recommendations, starts in the first trimester, and continues throughout pregnancy alongside usual care. It consists of individualized counseling sessions with an RD using motivational interviewing and electronic educational and support resources. This study will highlight the efficacy of such an intervention and its acceptability and feasibility in a population of pregnant individuals at risk for GDM.

This RCT will convincingly address the needs identified in the latest Diabetes Canada guidelines and will enrich the sparse literature on the matter. Currently, some studies have evaluated the effects of a nutritional intervention among pregnant individuals at risk for GDM but seemed hindered by substantial limitations, making it impossible to answer our research question. Moreover, none were conducted among Canadian populations. This study will offer valuable insights into diet quality, eating behavior, and quality of life, as well as their relation to the glycemic profile and incidence of GDM. We will also document several feasibility measures including recruitment rates, attrition rates, effective recruitment and retention strategies among this population, as well as the acceptability of the provided intervention.

The primary expected benefit for the individuals at risk receiving the intervention is improved glucose homeostasis that may lead to healthier pregnancy outcomes, reducing the need for medical interventions and associated complications while potentially decreasing the costs associated with those interventions. Hence, the early nutritional intervention provided could be beneficial for a significant number of individuals who may not have access to or benefit from the services of an RD otherwise. By focusing on prevention and empowerment, this intervention aims to improve the overall well-being of pregnant individuals at risk for GDM and contribute to their long-term health and the health of their newborns.

The evidence generated by this trial will also benefit various stakeholders. Perinatal care professionals such as RDs, nurses, obstetricians, and endocrinologists could potentially benefit from generated evidence-based data within this trial to enhance the quality of care they provide. Additionally, the study’s results may also highlight the importance of integrating web-based resources and virtual support communities in nutritional interventions, potentially paving the way for innovative and scalable approaches to healthcare delivery. To this end, collaborations with healthcare professionals will facilitate the direct application of the trial results in their clinical practice. Health agencies and policymakers can benefit from the RCT’s findings as well, which may lead to the development of evidence-based guidelines and policies for managing GDM risk in pregnant individuals. Integrating such interventions into standard antenatal care could improve pregnancy outcomes on a larger scale and reduce the burden on healthcare systems. Overall, the trial results will inform and improve clinical practice, enhance patient care, and guide decision-making processes for healthcare stakeholders involved in managing GDM during pregnancy.

This RCT presents some limitations. The use of multiple self-reported data and the repetition of some questionnaires during participation may increase the risk of bias. However, the use of a web-based platform for self-completion of questionnaires may reduce the desirability bias. In addition, the repetition of the dietary questionnaires allows us to characterize more precisely the dietary habits and behaviors of the participants from their first trimester of pregnancy until after their delivery. Moreover, these biases should be similar in the intervention and control groups. As for any RCT, participant non-compliance with the assigned intervention or drop out from the study can affect the integrity and validity of the results and this study may not capture the complexity and nuances of real-world settings as the research conditions may differ from routine clinical practice. We find that the use of complete case intention-to-treat analyses, combined with weighted sensitivity analyses assessing the effect of treatment adherence may limit these potential challenges.

Despite its limitations, this trial presents some notable strengths. First, the measurement of multiple parameters related to glucose metabolism and lifestyle factors (diet, physical activity, and anxiety) enables precise characterization of the health status of participants and their offspring and birth, and their associations with maternal factors. The longitudinal follow-up included in this RCT also enables the evaluation of sustained effects and the assessment of outcomes over an extended period. Finally, the planned study relies on the active collaboration of local physicians and researchers, patient partners, and influential decision-makers. This collaborative approach enhances the potential impact and use of the study’s findings. The results of this trial are poised to have a significant scientific impact, as they will furnish essential insights into the significance of nutrition care designed to support pregnant individuals at risk for GDM. We anticipate that this study will improve perinatal outcomes, optimize glucose tolerance, and contribute to healthier pregnancies. Consequently, the SAGE study holds the potential to enhance the antenatal care of individuals at risk for GDM while maintaining an acceptable and feasible approach.

In this paper, we present the rationale and methodology of an RCT that will assess the effects of early nutritional intervention on the glycemic parameters of pregnant individuals at risk for GDM. This study will offer valuable insights into the potential of interventions aiming at improving glucose homeostasis based on current national recommendations. If the intervention demonstrates favorable glucose homeostasis markers throughout pregnancy, steps will be taken to integrate it into the follow-up of at-risk pregnant individuals. Larger-scale trials will also be needed to evaluate the intervention’s effects on maternal and fetal outcomes during pregnancy. Ultimately, if found to be effective, this intervention could have the potential to be incorporated into routine clinical practice, benefiting pregnant individuals at risk for GDM and their children. This project will thus contribute to the growing body of evidence and help inform future recommendations and guidelines for nutritional interventions targeting individuals at risk for GDM during pregnancy, to optimize the management of pregnant individuals at nutritional and metabolic risk, and emphasize the importance of adopting healthy eating habits early in pregnancy.

### Dissemination

The findings of the study will be disseminated through presentations at provincial, national, and international conferences, as well as publication in peer-reviewed journals. Additionally, the results will be made accessible to the public through publication on clinicaltrials.gov. The presented and published results will ensure the anonymity of study participants and will not allow for their identification.

## Data availability statement

The original contributions presented in the study are included in the article/Supplementary material, further inquiries can be directed to the corresponding author.

## Ethics statement

The studies involving humans were approved by Ethics Committee of the Centre de recherche du CHU de Québec-Université Laval, Québec, QC, Canada. The studies were conducted in accordance with the local legislation and institutional requirements. The participants provided their written informed consent to participate in this study.

## Author contributions

EB: Conceptualization, Methodology, Writing – original draft, Writing – review & editing. A-SP: Conceptualization, Methodology, Writing – review & editing. PL: Conceptualization, Methodology, Writing – review & editing. JR: Conceptualization, Methodology, Writing – review & editing. SL: Writing – review & editing. SoD: Conceptualization, Methodology, Writing – review & editing. AB-G: Writing – review & editing. SM-L: Writing – review & editing. SW: Conceptualization, Methodology, Writing – review & editing. SuD: Writing – review & editing. FC-L: Formal Analysis, Methodology, Writing – review & editing. MB: Conceptualization, Methodology, Writing – review & editing. J-PB: Conceptualization, Formal Analysis, Methodology, Writing – review & editing. A-SM: Conceptualization, Formal Analysis, Funding acquisition, Investigation, Methodology, Writing – review & editing.
