# Supplementary material for: Promoting healthy eating in early pregnancy in individuals at risk of gestational diabetes mellitus: does it improve glucose homeostasis? A study protocol for a randomized control trial
Source: Front Nutr. 2024 Jan 19;10:1336509. doi: 10.3389/fnut.2023.1336509 (PMC10834641; doi:10.3389/fnut.2023.1336509)
Supplement: Supplementary file 1 [file Data_Sheet_1.docx]

Supplementary Material

# Supplementary Data

**The development of the video clips has been done in a rigorous way:**

Step 1: A first proposal of themes, objectives and content was elaborated by a graduate student supervised by the PI.

Step 2: Following the research team’s discussion, themes and content has been proposed to the co-investigators and then to the advisory committee of experts (research team collaborators comprised of 1 endocrinologist treating patients with GDM (SJW), 1 clinical RD (MB), 2 RDs, also researchers with expertise in knowledge transfer (SD) and in interventions studies in that population (JD).

Step 3: Themes and content were modified according to comments received and a first draft of the verbatim was developed for each web clip. The verbatim were reviewed by communication experts (Préambule).

Step 4: Changes have been made accordingly, and visuals media were developed to accompany the verbatim, with the support of a graphic design expert.

Step 5: Before recording, the visuals media and verbatim was presented in the form of a formal presentation (in person or virtual) to all members of the *provincial Round-Table of registered dietitians working in maternal-fetal medicine* and to a group of endocrinologists.

Step 6: Changes were then made, and professional audio-recording was performed.

Step 7: The final editing, with the addition of a visual introduction and conclusion, was done by a graphic expert. The videos are hosted on private YouTube chain and will be shared with participants in the intervention group.

# Supplementary Tables

**Table S1:** Video clips content summary

| **Video clips** | **Themes** | **Aims** |
| --- | --- | --- |
| 1 | Importance of healthy eating during pregnancy | 1. To demonstrate the importance and benefits of healthy eating during pregnancy on the short- and long-term health of the mother and child. 2. To instill awareness of pregnancy discomforts and complications. 3. To get participants to think about what their motivations are to improve their diet and to reflect on possible daily improvements. |
| 2 | Should we eat differently during pregnancy? | 1. To describe the specific physiological changes and dietary needs during pregnancy. 2. To get participants to reflect on possible changes they should make to meet their needs. |
| 3 | Canadian Food Guide recommendations and balanced meal examples | 1. To present the Canadian Food Guide (CFG) balanced plate and key messages. 2. To get participants to reflect on possible changes they should make to meet those recommendations. |
| 4 | Food safety  during pregnancy | 1. To present the food choices to avoid during pregnancy and the associated risks. 2. To state the necessary precautions to avoid infections and food poisoning during pregnancy. 3. To raise awareness about the consequences of food poisoning and alcohol consumption on pregnancy outcomes and the baby’s health. |
| 5 | Favorable habits to adopt around the act of eating | 1. To present favorable habits to adopt around the act of eating and related benefits. 2. To get participants to reflect on their habits around the act of eating and changes they could make. |
| 6 | Why and how to choose low-processed foods? | 1. To define what is a highly, minimally and unprocessed foods. 2. To demonstrate the risks associated with the consumption of highly processed foods and the benefits of limiting them. 3. To get participants to recognize common processed foods in their diet. 4. To get participants to think about ways to limit their processed foods intake and replace them by healthier options. |
| 7 | Marketing and food labelling’s influence | 1. To define food marketing. 2. To present the most common food marketing strategies and how they can influence the food choices. |
| 8  (optional) | Discomforts during pregnancy: what is the role of nutrition? | 1. To present dietary strategies to reduce the discomfort of pregnancy symptoms. |
| 9 (optional) | Nutrients for a healthy diet | 1. To define and explain the basic roles of the macronutrients. 2. To get participants to balance macronutrients in their diet. |
| 10 (optional) | How to read nutrition labels? | 1. To explain how to read a nutrition label. 2. To get participants to use nutrition labels to make better food choices. |

**Table S2:** Video clips detailed content

| **Video clip 1** | | | | | | | | | | | | |
| --- | --- | --- | --- | --- | --- | --- | --- | --- | --- | --- | --- | --- |
| Theme | | | | | Importance of healthy eating during pregnancy | | | | | | | |
| Duration | | | | | 4:13 | | | | | | | |
| Aims | | | | | 1. To demonstrate the importance and benefits of healthy eating during pregnancy on the short- and long-term health of the mother and child. 2. Be aware of pregnancy discomforts and complications. 3. To get participants to think about what their motivations are to improve their diet and to reflect on possible daily improvements. | | | | | | | |
| Content | | | | | - Benefits of healthy eating during pregnancy on both mother and child (1,2,3). A healthy diet during pregnancy can:   - Contribute the baby’s growth and development (1,2,3,4).   - Provide the essential vitamins, minerals and other nutrients needed by both the mother and child (1,2,4).   - Mother’s energy during pregnancy (1).   - Contribute to a healthy weight gain (1).   - Influence the global health of the unborn baby (2,3,4).   - Promote a healthy pregnancy (2,4).   - Promote the adoption of some healthy eating behaviors and food choices later on during the child’s life (3).   - Prevent or reduce some of the discomforts of pregnancy (4). - Introduction of the main complications and side effects during pregnancy that might impact nutrition (2,4). *Refer interested participants to* ***Video clip 8.***   - For example, food cravings and aversions, bloating, heartburn and reflux, nausea and vomiting, constipation, fatigue, insomnia. - Introduction of the Canadian Food Guide Plate as an example of healthy eating (5). *Refer participants to* ***Video clip 3.*** | | | | | | | |
| Visuals | | | | | 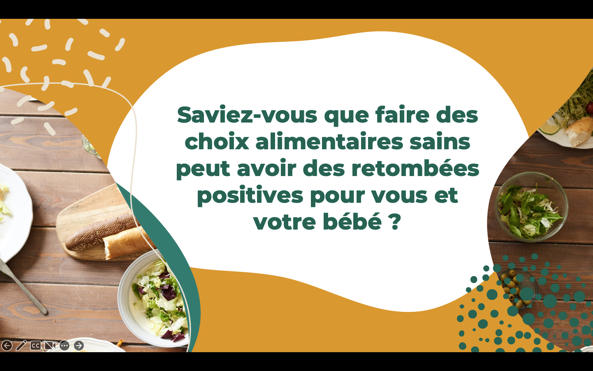 | | | | | | 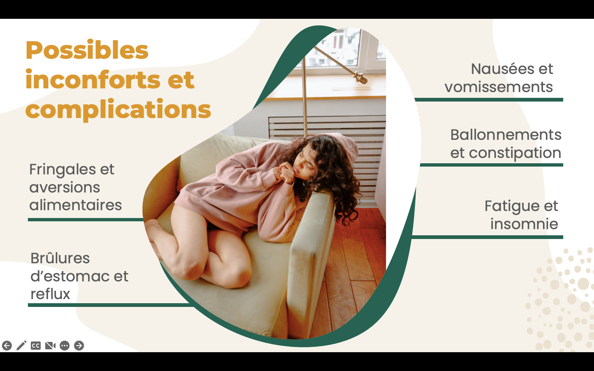 | |
|  |  |  |  |  | 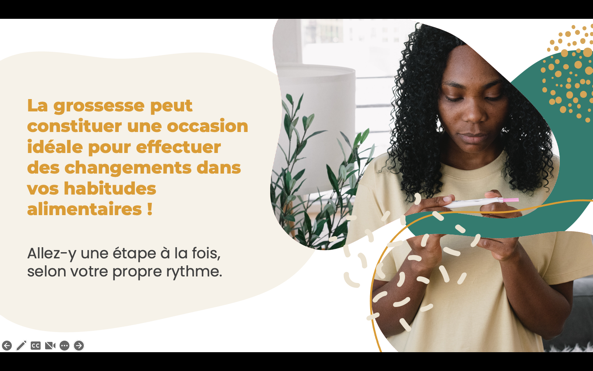 | | | | | | 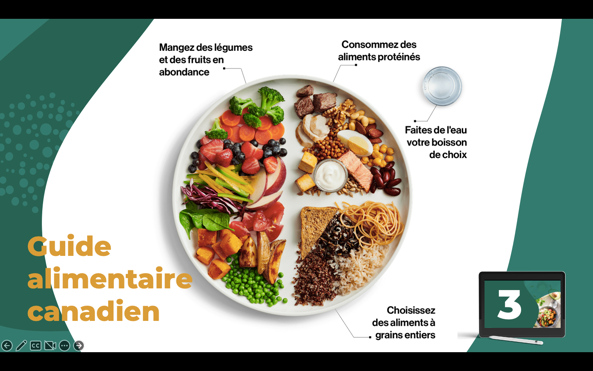 | |
| References | | | | | 1. Gouvernement du Canada. (2021) *Guide alimentaire canadien, Une saine alimentation pendant la grossesse et l’allaitement.* <https://guide-alimentaire.canada.ca/fr/conseils-pour-alimtation-saine/grossesse-allaitement/>  2. Gouvernement du Canada. (2021) *Votre guide pour une grossesse en santé.* Agence de la santé publique du Canada <https://www.canada.ca/fr/sante-publique/services/promotion-sante/grossesse-sante/guide-grossesse-sante.html>  3. Fondation OLO (2020) *Avoir un enfant : une bonne raison d’améliorer son alimentation* <https://fondationolo.ca/blogue/alimentation/avoir-un-enfant-une-bonne-raison-ameliorer-son-alimentation/>  4. Doré, N et Le Hénaff, D. (2020) *Mieux vivre avec notre enfant de la grossesse à deux ans : guide pratique pour les parents.* Québec, Institut national de santé publique du Québec.  5. Gouvernement du Canada. (2021) *Guide alimentaire canadien* <https://guide-alimentaire.canada.ca/fr/> | | | | | | | |
| **Video clip 2** | | | | | | | | | | | | |
| Theme | | | | | | Should we eat differently during pregnancy? | | | | | | |
| Duration | | | | | | 8:23 | | | | | | |
| Aims | | | | | | 1. To describe the specific physiological changes and dietary needs during pregnancy. 2. To get participants to reflect on possible changes they should make to meet their needs. | | | | | | |
| Content | | | | | | - Physiological needs of the mother and the baby during pregnancy:   - During pregnancy, individuals have special dietary needs, namely because of the physical changes that increase the mother's need for fluids and nutrients (1,2,3). - Food intakes:   - Eat regularly, at least three meals a day, according to your appetite (4,5).   - Eat a variety of foods by composing meals with vegetables, fruits, whole grain products and protein foods (1,2,3,4,5). *Refer to* ***Video clip 3.***   - Restrictive dieting should be avoided during pregnancy (1,3).   - During the second and third trimesters, the mother needs to eat more daily to support the baby’s development (1,2,3).     - The addition of a snack, a light meal or bigger portion sizes are thus indicated to meet the mother and baby’s growing needs (1,2,3). - Fluid requirements:   - Increased requirements by 50% during pregnancy (1,3).   - Make water your drink of choice (1,2,5).   - Benefits of water over other beverages: cooling action, reduces swelling, prevents constipation, headaches and fatigue, removes waste products, transports nutrients (1,2,3).   - Drink water as often as possible and supplement with nutritious fluids such as milk and fortified soy beverages (1,2,3,5). - Essential nutrients:   - Iron, folic acid, calcium, and vitamin D:     - Pregnancy significantly increases your need for several nutrients, such as iron and folic acid (1,2,3).     - It is recommended to take a multivitamin containing folic acid from two or three months prior to pregnancy to delivery (1,2,3,4).     - The prenatal multivitamin should contain at least 0.4 mg of folic acid and 16-20 mg of iron (1,2,3).     - For women who don't consume much dairy or fortified soy beverages, the chosen multivitamin should also contain calcium and vitamin D (3).   - Healthy fats (omega-3s):     - Pregnant women need more omega-3 fatty acids to support the growth of the baby's brain and tissues (1,2,3,5).     - Choose foods that contain them such as nuts, seeds, low-mercury fatty fish or vegetable oils (1,2,3,5). Some species of fish contain contaminants, such as mercury. Pregnant women should consume fish chosen with care. Refer to ***Video clips 4.***     - Eating two fatty fish meals a week provides the recommended intakes of omega-3s (3). - Fiber:   - - Found in several food categories: whole grain foods, vegetables and fruits, legumes, nuts and seeds (3). *Refer to* ***Video clip 9.***     - Help prevent constipation (3). *Refer to* ***Video clip 8.*** | | | | | | |
| Visuals | | | | | | 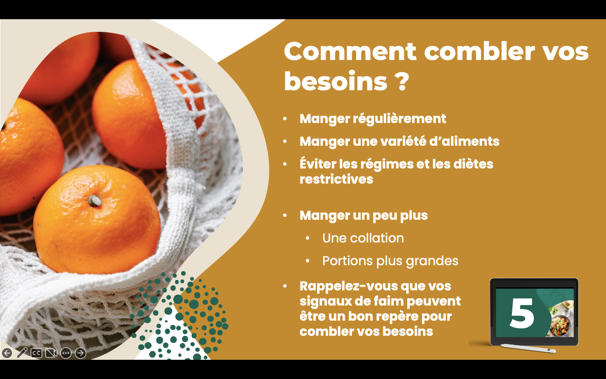 | 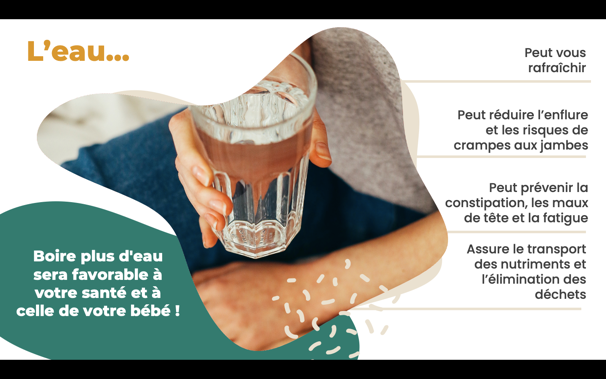 | | | | | |
|  |  |  |  |  |  | 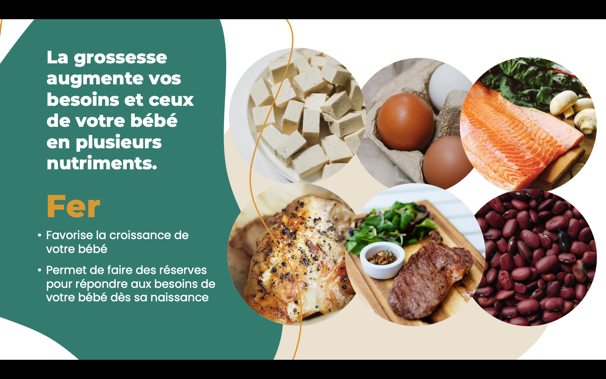 | 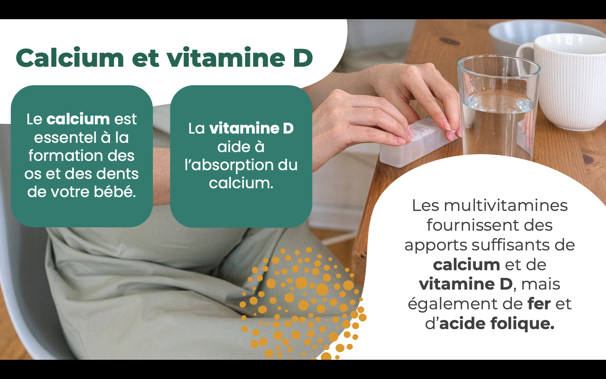 | | | | | |
| References | | | | | | 1. Gouvernement du Canada. (2021) *Guide alimentaire canadien, Une saine alimentation pendant la grossesse et l’allaitement.* <https://guide-alimentaire.canada.ca/fr/conseils-pour-alimtation-saine/grossesse-allaitement/>  2. Gouvernement du Canada. (2021) *Votre guide pour une grossesse en santé.* Agence de la santé publique du Canada <https://www.canada.ca/fr/sante-publique/services/promotion-sante/grossesse-sante/guide-grossesse-sante.html>  3. Doré, N et Le Hénaff, D. (2020) *Mieux vivre avec notre enfant de la grossesse à deux ans : guide pratique pour les parents.* Québec, Institut national de santé publique du Québec.  4. Fondation OLO (2018) *Je suis enceinte : quoi faire?* <https://fondationolo.ca/blogue/alimentation/bien-manger/je-suis-enceinte-quoi-faire/>  5. Gouvernement du Canada. (2021) *Guide alimentaire canadien* https://guide-alimentaire.canada.ca/fr/  Supplemental references :  <https://fondationolo.ca/blogue/grossesse/tout-savoir-sur-le-fer-pendant-la-grossesse/> | | | | | | |
| **Video clip 3** | | | | | | | | | | | | |
| Theme | | | | | | Canadian Food Guide recommendations and balanced meal examples | | | | | | |
| Duration | | | | | | 12:26 | | | | | | |
| Aims | | | | | | 1. To present the Canadian Food Guide (CFG) balanced plate and key messages. 2. To get participants to reflect on possible changes they should make to meet those recommendations. | | | | | | |
| Content | | | | | | - Presentation of the balanced plate:   - Proportions rather than portions (1).   - Reiterate the importance of healthy eating during pregnancy and after. *Refer to* ***Video clip 1*.**     - Each food group gives the mother and child different essential nutrients that are needed during pregnancy (2,3).     - Provide the mother with the energy she needs, ensures baby's development, maintains good health for both mother and child, promote healthy weight gain and healthy pregnancy outcomes (2,3). - Presentation of the key messages:   - Eat plenty of vegetables and fruit, whole grains and protein foods (1,2).     - As often as possible, create balanced meals or snacks by combining these foods.     - Vegetables and fruits:   - Should represent 50% of the food on your plate (1,4). Fresh, frozen and canned vegetables and fruit are all healthy choices (1).  - Make sure to wash the vegetables or fruits before consumption. *Refer to* ***Video clip 4***.   - - - Protein foods:   - Should represent 25% of the food on your plate (1).  - Why and how to choose plant-based protein foods more often (1).   - - - Whole grain foods:   - Should represent 25% of the food on your plate (1).  - Whole grain foods contain more fiber than refined grain foods (1). Introduce the benefits of fiber (i.e.: constipation). *Refer to* ***Video clip 3****.*   - - Make water your drink of choice by replacing sugary drinks with water (1,2,3,4).     - Introduce the benefits of water over other beverages. *Refer to* ***Video clip 2***.   - Choose foods with good fats instead of saturated fats, namely omega-3s (1,4). *Refer to* ***Video clip 2***.   - Use ingredients that have little or no added sodium and sugars (1,4).   - Limit highly processed foods. *Refer to* ***Video clip 6***. If you do choose them, eat them less often and in smaller amounts (1). - Examples will be given for each food group and recommendations:   - Examples of plates with other types of foods and from different cultures. | | | | | | |
| Visuals | | | | | | 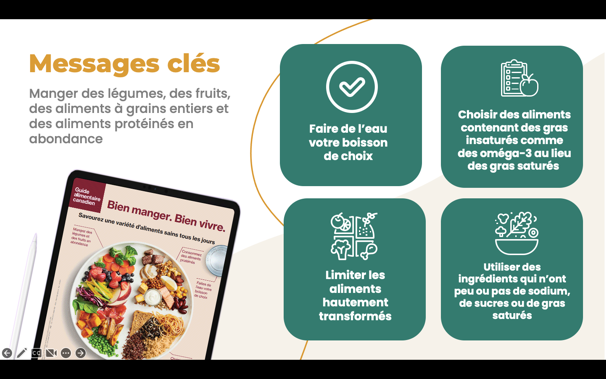 | 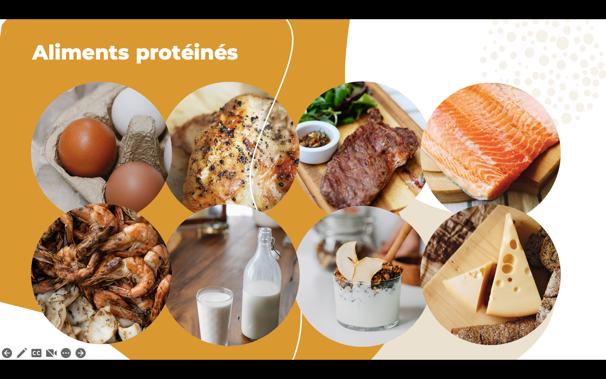 | | | | | |
|  |  |  |  |  |  | 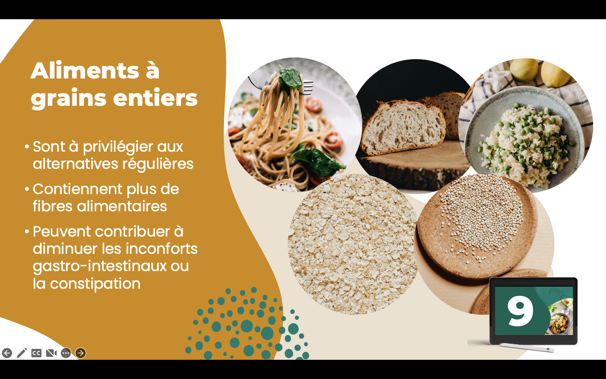 | 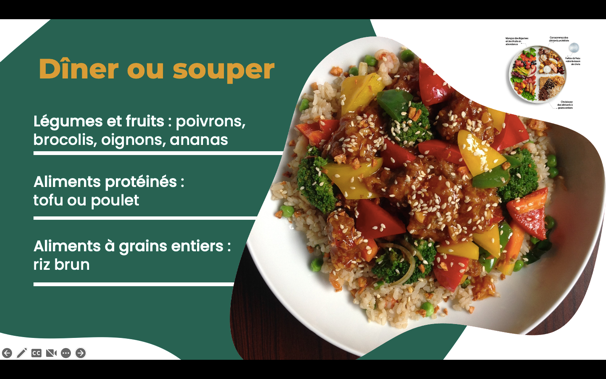 | | | | | |
| References | | | | | | 1. Gouvernement du Canada. (2021) *Guide alimentaire canadien* <https://guide-alimentaire.canada.ca/fr/>  2. Gouvernement du Canada. (2021) *Guide alimentaire canadien, Une saine alimentation pendant la grossesse et l’allaitement.* <https://guide-alimentaire.canada.ca/fr/conseils-pour-alimtation-saine/grossesse-allaitement/>  3. Doré, N et Le Hénaff, D. (2020) *Mieux vivre avec notre enfant de la grossesse à deux ans : guide pratique pour les parents.* Québec, Institut national de santé publique du Québec.  4. Gouvernement du Canada. (2021) *Votre guide pour une grossesse en santé.* Agence de la santé publique du Canada. <https://www.canada.ca/fr/sante-publique/services/promotion-sante/grossesse-sante/guide-grossesse-sante.html>  Supplemental references: <https://www.jaimefruitsetlegumes.ca/fr/blogue/manger-plus-de-fruits-et-legumes-une-portion-a-la-fois-un-repas-a-la-fois/> and <https://fondationolo.ca/blogue/alimentation/les-aliments-a-grains-entiers/> | | | | | | |
| **Video clip 4** | | | | | | | | | | | | |
| Theme | | Food safety during pregnancy | | | | | | | | | | |
| Duration | | 13:32 | | | | | | | | | | |
| Aims | | 1. To present the food choices to avoid during pregnancy and the associated risks. 2. To state the necessary precautions to avoid infections and food poisoning during pregnancy. 3. To raise awareness about the consequences of food poisoning and alcohol consumption on pregnancy outcomes and the baby’s health. | | | | | | | | | | |
| Content | | - Importance of food safety during pregnancy:   - In response to all the physiological changes during pregnancy, the mother’s immune system is weaker (1,2,3,4).   - The unborn baby's immune system is not developed enough to fight off dangerous bacteria which can cross the placenta (3).   - She and her unborn baby are at greater risk of contracting infections (1,2,3).   - Thus, the risk of food poisoning increases when pregnant (1,2,3).   - Food poisoning can be dangerous for the mother and ever more serious for the unborn baby (1,3).     - Complications: miscarriage, preterm birth, malformation, baby’s global health at birth, etc. (3,4).   - “Because your baby depends on you for everything, it is very important to pay attention to your diet and how you store, prepare and cook food” (3).     - Some foods need to be temporarily avoided during pregnancy (1,2,3,5,6,7). - Food choices to avoid during pregnancy to limit food poisoning:   - Certain types of food may pose a higher risk to pregnant women because of the way they are produced and stored (3).   - Risk of food poisoning can be decreased by avoiding the following foods (2,3,4,6,7):     - Raw or unpasteurized dairy products;     - Raw or undercooked meat, poultry and seafood;     - Liver;     - Raw fish such as sushi, tartar, ceviche, oysters, clams and mussels and refrigerated smoked seafood such as smoked salmon;     - Unpasteurized juices, such as unpasteurized apple cider and kombucha;     - Unwashed fruits and vegetables;     - Raw sprouts, such as alfalfa, clover, radish and mung beans;     - Hot dogs taken directly from the package without cooking;     - Uncured deli meats such as bologna, roast beef, ham and turkey breast;     - Refrigerated pâté, refrigerated meat spreads and refrigerated smoked seafood;     - Raw or lightly cooked eggs (sunny-side up, soft-boiled or poached eggs or egg products that contain raw eggs (such as homemade Caesar dressing, cookie dough, cake batter, sauces, eggnog, mousses);     - The following pasteurized and unpasteurized cheeses: soft cheeses (such as Brie, Camembert, Bocconcini, Feta), semi-soft cheeses (such as Havarti and Saint-Paulin), blue cheeses (such as Roquefort and Stilton) and cheeses made from raw milk. - Habits to adopt during pregnancy to limit food poisoning:   - Food storage and use:     - Refrigerate perishable foods as soon as you return from the grocery store (3).     - Maintain proper storage temperature (Set your refrigerator at 4°C (40°F) or lower and your freezer at -18°C (0°F) or lower) (3).     - Respect the "best before" date on refrigerated perishable products (2).   - Before cooking:     - Wash your hands with soap before handling food (2,4).     - Clean utensils, plates, cutting boards, counters and work surfaces before handling food (2,4).     - Rinse your vegetables and fruit with water before cutting or eating them, raw or cooked, with or without peel (2,4).     - Thaw food safely (i.e., in the refrigerator or microwave) (2,3,4).   - While cooking:     - Use clean surfaces and equipment (4).     - Avoid cross-contamination (4).     - Cook food safely (4). To find out if food is cooked safely, you can use a digital food thermometer and check the internal temperature (2).   - After cooking:     - Refrigerate or freeze your table scraps quickly and use them as soon as possible (3,4). Don't refreeze any food unless you have cooked it after thawing (2).     - Rewash your hands with soap after handling food (2,4).     - Clean utensils, plates, cutting boards, counters and work surfaces after handling food (2,4).     - Change or wash your kitchen clothes regularly (2). - Other food choices to limit or avoid during pregnancy:   - Caffeine     - Caffeine is safe when consumed in small amounts but should thus be limited to 300 mg per day, which is about two 8-ounce (237 ml) cups of coffee or three espressos (30 ml) (1,2,5,6,7).     - Caffeine is also found in other beverages such as teas, kombucha, soft drinks, energy drinks and chocolate (1,2,5,6,7).   - Herbal teas     - Some herbal products can interfere with the pregnancy, by causing contractions, for example (2).     - The following herbal teas are generally safe if consumed in moderation (no more than 2-3 cups per day): orange or other citrus peel, ginger, lemon balm, rosehips, linden blossom (not recommended if heart problems) (2,7).     - Some teas or blended teas contain ingredients that are not recommended during pregnancy.     - These should be avoided: chamomile tea, aloe vera leaves, coltsfoot, juniper berries, spearmint, buckthorn bark, comfrey, labrador tea, sassafras, patience root, lobelia and senna leaves, etc. (2,7).   - Fish with high mercury content:     - For the baby's development, it is encouraged to eat well-cooked healthy fatty fish (e.g., trout, salmon, mackerel or sardines) (1,2,7).     - It is recommended to put aside large fish that contain a lot of mercury, which can affect your baby's brain development (such as tuna (*Note that canned light tuna is safe), shark, swordfish, marlin, orange roughy and escolar etc.) (1,2,7,8). - Alcohol consumption:   - Alcohol is a teratogen, which means it can harm your baby's development (6).   - Since the placenta does not filter alcohol, it’s carried out to blood stream the unborn baby (2,8).   - Alcohol can have several harmful effects during and after the pregnancy: it can cause malformations, miscarriage, premature delivery or stillbirth but can cause brain damage to the baby resulting in learning or memory problems, attention deficit, problem-solving difficulties and behavioral problems later in life (such as the Fetal Alcohol Spectrum Disorder which causes permanent disability) (2,6,8).   - Thus, throughout pregnancy, it is recommended not to drink alcohol (2,6,8).     - The more alcohol consumed, the greater the risk of damage to the baby. Drinking a large amount of alcohol on one occasion and regular drinking are particularly harmful (2).     - The effect of alcohol on the baby is the same no matter what type of drink you drink: beer, wine or hard liquor (2). | | | | | | | | | | |
| Visuals | | 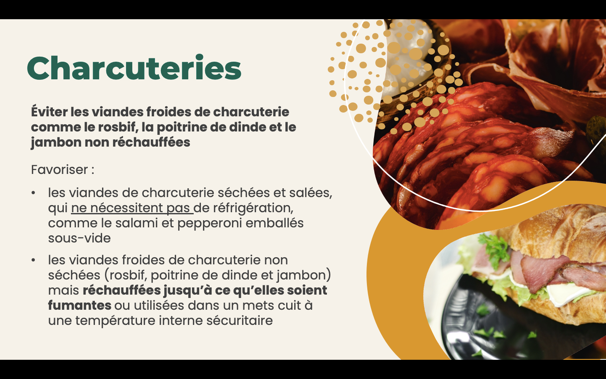 | | | | | | | | 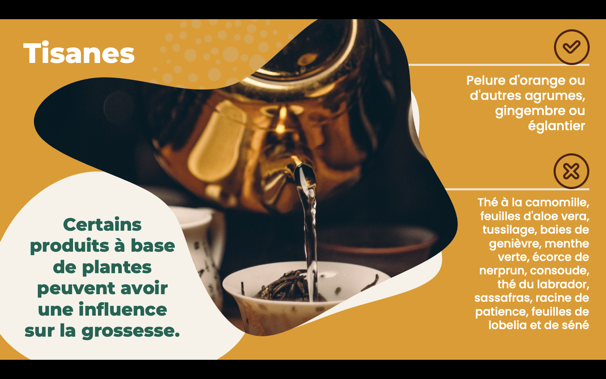 | | |
|  |  | 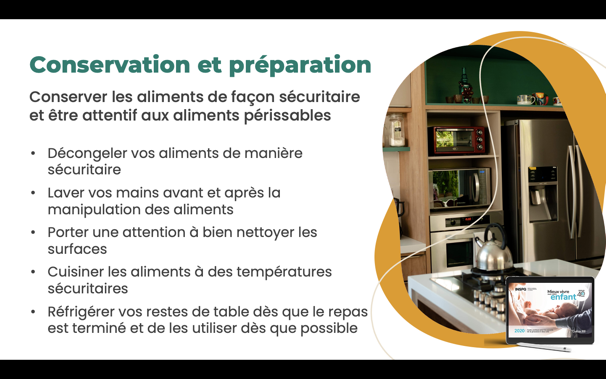 | | | | | | | | 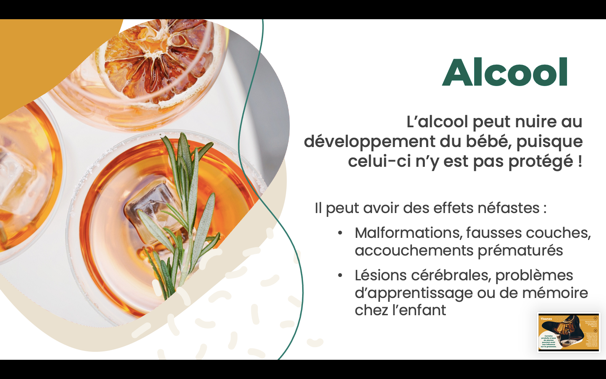 | | |
| References | | 1. Gouvernement du Canada. (2021) *Guide alimentaire canadien, Une saine alimentation pendant la grossesse et l’allaitement.* <https://guide-alimentaire.canada.ca/fr/conseils-pour-alimtation-saine/grossesse-allaitement/>  2. Doré, N et Le Hénaff, D. (2020) *Mieux vivre avec notre enfant de la grossesse à deux ans : guide pratique pour les parents.* Québec, Institut national de santé publique du Québec.  3. Gouvernement du Canada. (2019) *Salubrité des aliments pour les populations vulnérables, La salubrité des aliments pour les femmes enceintes.* <https://www.canada.ca/fr/sante-canada/services/salubrite-aliments-pour-populations-vulnerables/salubrite-aliments-pour-femmes-enceintes.html>  4. Fondation OLO (2021) *Comment prévenir l’intoxication alimentaire?* <https://fondationolo.ca/blogue/dossiers/hygiene-et-salubrite/comment-prevenir-lintoxication-alimentaire/>  5. Fondation OLO (2018) *Je suis enceinte : quoi faire?* <https://fondationolo.ca/blogue/alimentation/bien-manger/je-suis-enceinte-quoi-faire/>  6. Gouvernement du Canada. (2021) *Votre guide pour une grossesse en santé.* Agence de la santé publique du Canada.  <https://www.canada.ca/fr/sante-publique/services/promotion-sante/grossesse-sante/guide-grossesse-sante.html>  7. Fondation OLO (2019) *Quoi manger et ne pas manger enceinte?* <https://fondationolo.ca/blogue/grossesse/quoi-ne-pas-manger-enceinte/>  8. Fondation OLO (2019) *Alcool et grossesse : un peu, un tout petit peu ou pas du tout?* <https://fondationolo.ca/blogue/grossesse/alcool-et-grossesse/>  Supplemental references :  <https://www.dispensaire.ca/articles/risques-associes-consommation-dalcool-grossesse/>  <https://www.inspq.qc.ca/information-perinatale/fiches/alcool> | | | | | | | | | | |
| **Video clip 5** | | | | | | | | | | | | |
| Theme | | | | Favorable habits to adopt around the act of eating | | | | | | | | |
| Duration | | | | 9:07 | | | | | | | | |
| Aims | | | | 1. To present favorable habits to adopt around the act of eating and related benefits. 2. To get participants to reflect on their habits around the act of eating and changes they could make. | | | | | | | | |
| Content | | | | Healthy eating is more than just the food you eat!   - Be more aware of your eating habits (1,2,3).   - Definition: “Being aware of your eating habits means being aware of : what you eat, when you eat, where you eat, how you eat, why you eat, how much you eat” (1).   - Benefits: “Making healthier choices more often, make positive changes to your usual eating behaviors, be more aware of the foods you eat and your eating habits, become more aware of your daily eating decisions, and to reconnect with the experience of eating’ (1). - You can try to be more aware of your eating habits by:   - Taking time to eat (1,3):     - With busy lifestyles, it is common to: eat quickly, eat while distracted or doing something else, not setting aside time to eat and plan meals, which can all prevent you from realizing that you have eaten enough and can cause you to eat more than you need (1).     - Benefits of taking the time to eat: avoid overeating, enjoy your food, make healthier food choices, enjoy eating with others, focus on what you are eating, be aware of your eating habits and choices (1).     - How to make time to eat: Eat slowly and mindfully, focus on your food while you eat, eat without distractions, plan your meal times (1).   - Using your senses (1):     - Benefits: allows you to pay attention to the aromas, textures, flavors and taste, discover what you like and dislike through your senses, allows you to reconnect with the experience of eating and be more aware of the foods you eat (1).   - Savoring food (1,4):     - Benefits: savoring flavors, being open to discovering new foods, developing a healthy attitude towards food (1).     - How to savor food: enjoy your food by making choices that reflect your tastes, your culture, your budget and your lifestyle, try new foods, create a positive food environment (1).   - Recognizing the pleasure of eating (1,5):     - Even if pregnancy sometimes comes with certain inconveniences (*Refer to* ***Video clip 9***), eating should remain a pleasure! So choose your meals according to your taste and desires (sometimes strange) of the moment (5).     - The pleasure of eating can include: enjoying grocery shopping, socializing at mealtime, eating food you love, preparing and cooking food, growing or harvesting your own food, getting to know the people who grow or produce your food, involving others in meal planning, preparation and clean-up.   - Recognizing the feelings of hunger and fullness (1):     - How hungry or full you feel can depend on many things, such as body signals, the look, smell, thought or accessibility of the food, and emotional state. However, eating in response to factors other than your body's signals can be linked to overeating and excessive energy intake (1).     - Benefits of paying attention to and responding to feelings of hunger or fullness: helps decide when and how much to eat, avoid overeating.     - Recognizing the signs that tell you if you are hungry or full: Pay attention and ask yourself if you are really hungry, be aware that emotional eating can influence your food choices, pay attention to the feeling of fullness to help you know when you have eaten enough, give your body time to digest and feel full before you decide to eat again, consider your food environment and remember that you may be eating because of the accessibility of food rather than because you are hungry. - Cook more often (1):   - Benefits: consume minimally to not processed food products, cook foods that you and your family will enjoy and eat, save money by avoiding extra restaurant expenses, choose healthy ingredients, learn new skills, discover new recipes, involve other family members in planning, meal prepping and cooking, encourage the passing of traditions (1).   - How to make cooking part of your routine: cook in large batches and freeze some meals; when you cook a dish, make enough to have for another meal; be creative and turn leftovers into a whole new meal; use time-saving appliances such as a slow cooker or food processor. (1) - Meal planning (1,2):   - Benefits: make healthier and loved choices, save time at the grocery store, prepare meals more quickly and without stress, reduce food waste by buying only what you need, save money (1).   - How to plan your meals in advance: set aside time to make your plan, make sure you have all the information you need regarding the recipes, make a grocery list (1).   - When creating your plan, consider: all your meals and snacks, your schedule, the amount of time you have to cook, the fresh, frozen, canned or dried ingredients you already have to reduce food waste and buy less (1). - Involving others in meal planning and preparation (1):   - Benefits: allows you to cook more often, builds healthy eating habits that will last a lifetime (including the skill of cooking healthy foods), allows you to pass on culinary and food traditions, spend valuable time with loved ones, learn and pass on important food skills, make meal and snack preparation more fun and less demanding, discover new and healthy foods that you would not normally try, build a stronger connection with those around you (1).   - How to get others involved in meal planning and preparation: invite a friend or relative over to cook some food, host a potluck or a cooking event, participate in food-related activities, participate in a community garden project, plan to go grocery shopping with a friend or neighbor to make this routine task more social, participate in a recipe exchange with your loved ones or colleagues (1). - Eat in the company of others (1):   - Benefits: enjoy quality time together, share and connect with others, discover new healthy foods that you might not normally try, pass on food traditions from one generation to the next and share them with other communities, and help your little one develops healthy eating behaviors (1).   - How: establishing a mealtime routine at home (4), planning lunches or dinners with others, organizing a series of dinners with friends, etc. (1). | | | | | | | | |
| Visuals | | | | 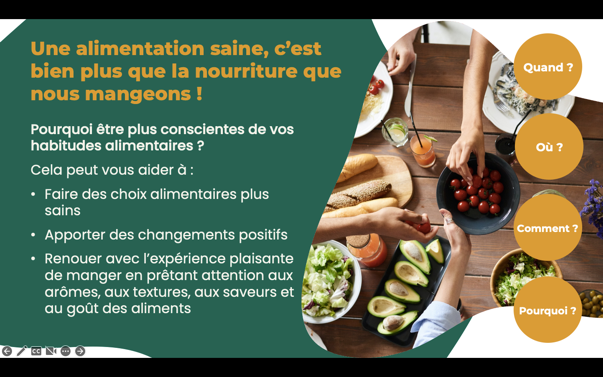 | | | | | | | | 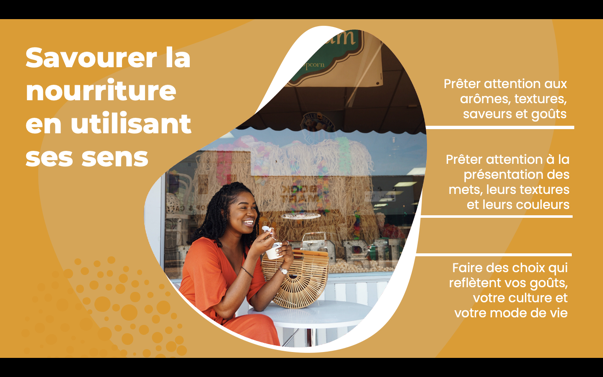 |
|  |  |  |  | 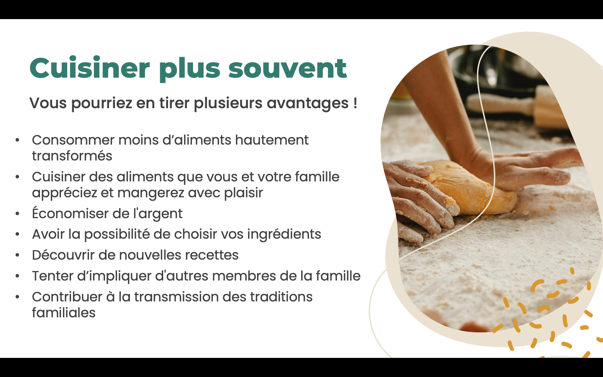 | | | | | | | | 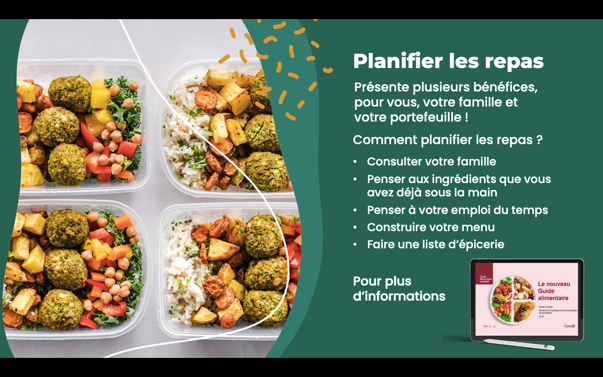 |
| References | | | | 1. Gouvernement du Canada. (2021) *Guide alimentaire canadien* <https://guide-alimentaire.canada.ca/fr/>  2. Gouvernement du Canada. (2021) *Guide alimentaire canadien, Une saine alimentation pendant la grossesse et l’allaitement.* <https://guide-alimentaire.canada.ca/fr/conseils-pour-alimtation-saine/grossesse-allaitement/>  3. Gouvernement du Canada. (2021) *Votre guide pour une grossesse en santé.* Agence de la santé publique du Canada. <https://www.canada.ca/fr/sante-publique/services/promotion-sante/grossesse-sante/guide-grossesse-sante.html>  4. Fondation OLO (2018) *Je suis enceinte : quoi faire?* <https://fondationolo.ca/blogue/alimentation/bien-manger/je-suis-enceinte-quoi-faire/>  5. Fondation OLO (2020) *5 trucs pour avoir une alimentation saine pendant la grossesse* https://fondationolo.ca/blogue/grossesse/alimentation-saine-grossesse/ | | | | | | | | |
| **Video clip 6** | | | | | | | | | | | | |
| Theme | | Why and how to choose low-processed foods? | | | | | | | | | | |
| Duration | | 9:53 | | | | | | | | | | |
| Aims | | 1. To define what is a highly, minimally and unprocessed foods. 2. To demonstrate the risks associated with the consumption of highly processed food products and the benefits of limiting them. 3. To get participants to recognize common processed foods in their diet. 4. To get participants to think about ways to limit their processed foods intake and replace them by healthier options. | | | | | | | | | | |
| Content | | - Food processing methods are essential for preservation and can even have a positive impact on the nutritional quality of foods (e.g., the addition of vitamin D to milk) (1). Other food processing methods, however, can have a negative impact on health because they increase the amount of fat, sugars and sodium in foods (1). - Risks associated to the consumption of highly processed foods:   - Regular consumption contributes to increased intakes of sodium, sugars and saturated fats which are linked to the increased risk of chronic disease when consumed in excess and should therefore not be consumed on a regular basis (1,4). - Benefits of limiting highly processed products:   - Eating less processed foods reduces the amount of fat, salt and sugars, which is compatible with higher diet quality and better health. - Whole foods:   - No processing involved.   - Examples: fresh fruits and vegetables, eggs, meat, poultry, fish, seafood, nuts, seeds and water (3). - General definition: A food that has been processed so that it is no longer in its raw or fresh form (2). Three types of processed foods: highly processed, processed, minimally processed. - Minimally processed foods:   - Food has undergone some type of processing that contributes to the safety of the food (e.g., pasteurization) or that helps preserve food and nutrients (e.g., drying, freezing, and canning) (3).   - Examples: frozen or dried fruits and vegetables, dried legumes, rice, pasta, couscous, flour, milk, etc. (3). - Processed foods:   - Food made from fresh or minimally processed foods by adding ingredients found in the kitchen (e.g., sugar, oil, salt or other ingredients) or by undergoing changes that affect little their nature, mainly to improve their conservation and nutritional quality, or to facilitate their preparation (2,3).   - Examples: canned foods (vegetables, fruits, legumes, fish...), fresh breads, cheeses, smoked meats and fish, salted or sweetened nuts/seeds, natural nut butters/seeds (peanuts, almonds...) (3). - Highly processed foods - To limit:   - Contain ingredients that are not found in the pantry such as additives or preservatives (3).   - High in sugar, fat and salt (3).   - Examples: fast foods, some breakfast cereals, flavoured oatmeal, some industrial breads, crackers, chips, salty snacks, cookies, candy bars, chocolate treats, pastries, muffins, cakes, candy, frozen meals (pizza, croquettes, pasta, etc.), sausages, cold cuts, instant soups or sauces, sweetened beverages (soft drinks, energy drinks, fruit cocktails, etc.) (2,3,4). - Ways to recognize the highly processed food products:   - Upon location in the grocery store (3):     - Often located in the aisles of the center and in the freezers (3).     - Caution: there are also interesting foods in these locations (e.g., frozen fruits and vegetables, canned legumes, rice and pasta, etc.) (3).   - Upon packaging (3). *Refer interested participants to* ***Video clip 10.***      - Ingredient list: Presence of long and complicated ingredient lists with words that are sometimes difficult to understand (3).     - Nutrition facts table: high in fat (saturated or trans fat), salt (sodium) and sugar as well as low fiber content (3).     - Presence of colorful and attractive packaging, sometimes containing characters known and loved by children that are designed to attract your attention (3).     - Don’t be fooled by logos or "health" claims (3):   - These claims may be true for one aspect of the food but, evaluated as a whole, the food may be less healthy (e.g., an ultra-processed food may be "high in calcium" or "high in fiber" but also contain a lot of sugar and fat.  - Warning: "Logos are created mainly by the food industry. You can't always trust them. They are based on a non-regulated categorization".   - How to limit highly processed foods:   - Cook more often (3,4). By cooking, you process the food yourself and use unprocessed or minimally processed foods (3). *Refer to* ***Video clip 5.***   - Choose foods that have no packaging, do not have children's characters (*Refer to* ***Video clip 7***), have a short ingredients list with ingredients that are easy-to-understand (no artificial ingredients) (2,3).   - Replace highly processed foods by healthier alternatives (4):     - Replace sugary drinks with water; Use leftover cooked chicken instead of deli products in sandwiches; Choose blunt oats instead of sweetened instant oatmeal; Make homemade versions of highly processed foods you like (i.e.: muffin, cookies, etc); Make your own frozen meals by choosing a healthy recipe to freeze in portions; Limit the addition of highly processed spreads and sauces to your meals and snacks.   - If you eat highly processed foods, try to eat them less often (in special occasions) and in smaller amounts. | | | | | | | | | | |
| Visuals | | 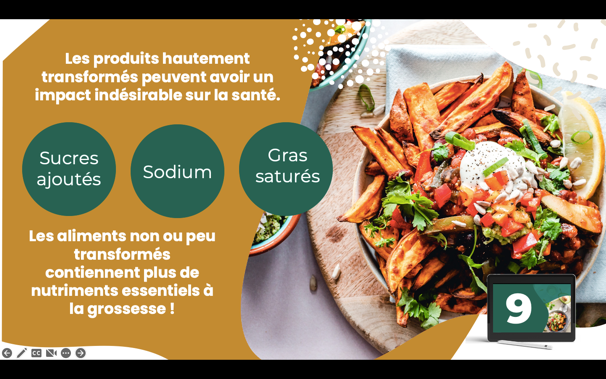 | | | | | | | | 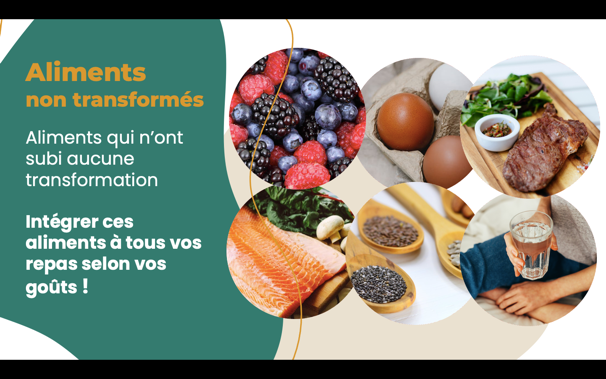 | | |
|  |  | 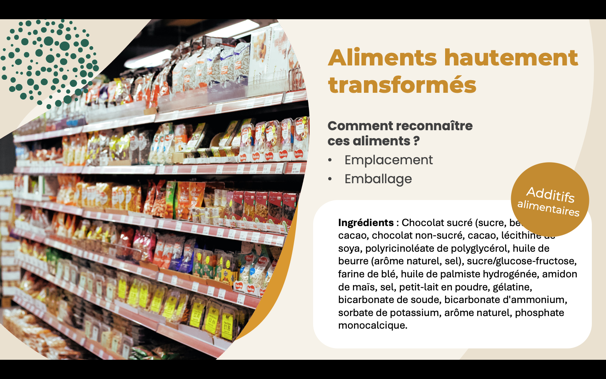 | | | | | | | | 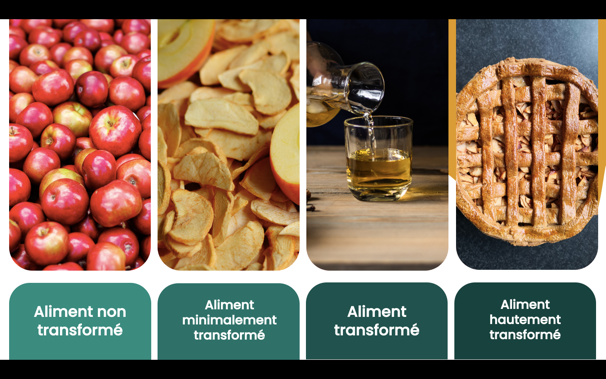 | | |
| References | | 1. Gouvernement du Québec (2019). *Aliments hautement transformés.* <https://www.quebec.ca/sante/alimentation/saines-habitudes-alimentaires/aliments-hautement-transformes>  2. Nos petits mangeurs (2015). *Quelle place pour les aliments transformés?* <https://www.nospetitsmangeurs.org/quelle-place-pour-les-aliments-transformes/>  3. Fondation OLO (2018). *Aliments ultra-transformés : les reconnaître et découvrir des alternatives.* <https://fondationolo.ca/blogue/alimentation/aliments-ultra-transformes-les-reconnaitre/>  4. Gouvernement du Canada. (2021) *Guide alimentaire canadien* <https://guide-alimentaire.canada.ca/fr/> | | | | | | | | | | |
| **Video clip 7** | | | | | | | | | | | | |
| Theme | | | Marketing and food labelling’s influence | | | | | | | | | |
| Duration | | | 6:37 | | | | | | | | | |
| Aims | | | 1. To define food marketing. 2. To present the most common food marketing strategies and how they can influence the food choices. | | | | | | | | | |
| Content | | | - Definition:   - Food marketing is a type of advertising that promotes selling certain foods or food products and may contribute to influencing our food choices (1,2).   - Food marketing can promote healthy foods such as fruits and vegetables but it is more often directed at ultra-processed foods. Thus, many marketed foods and beverages may be too high in sodium, sugars or saturated fats (1,2). *Refer to* ***Video clip 6****.*   - Food marketing can take many forms such as: branding, celebrity promotion, social media posts (sponsored or not, advertisements, product placements) (1).   - This influence often works without us realizing it. Almost like magic! But not quite. Food companies invest a lot of money in this field. They study our habits, our behaviors, our preferences, in short everything we do in relation to food, including adults and children (2).   - Marketing can also target you based on personal data such as age and gender, purchase history, Web browsing history (1). - How marketing influences choices (1):   - Food marketing is everywhere.   - Food marketing is designed to:     - Create food trends;     - Encourage you to buy certain foods or drinks;     - Create links between certain foods or brands and a particular lifestyle;     - Develop brand loyalty so that you will continue to purchase from a particular store or products with a brand name (1). - Common marketing strategies (2):   - Packaging (2):     - Bright colors and familiar characters are used to attract children.     - Healthy colors and images to appeal to parents!     - Convenient, easy-to-use formats which are not always economical.     - "Natural", "100% pure", "authentic"... and "health" logos: Beware that these claims, while they may be true, are not regulated or controlled. *Refer interested participants to* ***Video clip 10.***     - Claims such as "rich in calcium" or "low in cholesterol": These only show part of the quality of a product.   - Tip: On the package, only the nutrition facts table and the list of ingredients are presented with no sales intent. *Refer interested participants to* ***Video clip 10.***   - - Pricing (2):     - Weekly discounts, "3 for $5" promotions, coupons, etc.: These are only useful if they are not used to promote the product. They are useful only if they are necessary.   - Tip: Evaluate your real needs to avoid wasting food and money!   - - Displays (2):     - Eye-level products attract the eyes of young and old.     - Products at the end of the aisles.     - Products near the cash registers encourage impulse purchases, often unnecessary (candy bars, soft drinks, candy...).   - Tip: Make sure to look for advantageous options lower or higher in the aisles and to compare prices, nutrition facts table and ingredients lists.   - - Environment (2):     - Stimulation of the senses such as tastings or samples, smells, colors serve to awake your appetite! They encourage you to buy products that are often unnecessary and not always cheap.     - Basket sizes: The bigger it is, the more you may be tempted to fill it!   - Tip: Choose the basket size according to what you have to buy.   - How to stay vigilant other than tips stated above:   - Ask yourself why you want to buy a particular food or beverage. Is it good for you? Will it provide nutritional benefits? (1)   - Consider ways to reduce your exposure to advertising (1).   - Stick to your grocery list and avoid impulse buying (1).   - If you plan your meals and cook with basic foods, in addition to promoting healthy eating habits and saving money, you will outwit food marketing (2). *Refer to* ***Video clip 4****.*   - Go to the grocery store on a full stomach to limit temptation or impulse purchases. - Benefits of staying alert to food marketing:   - Recognize foods that are being marketed (1).   - Decide if a food is healthy by reading food labels rather than relying solely on marketing messages (1). *Refer to* ***Video clip 10****.*   - Educate people who may be more vulnerable to food marketing, such as young children and teens, about marketing techniques (1). | | | | | | | | | |
| Visuals | | | 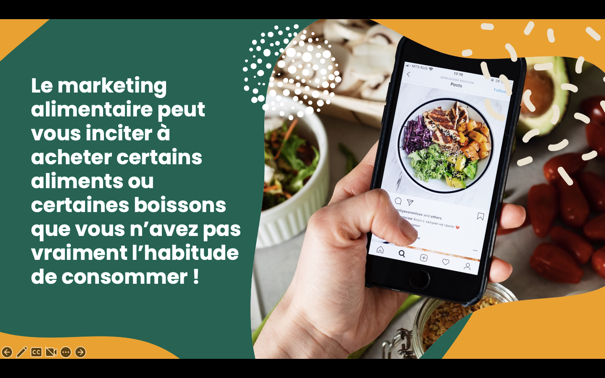 | | | | | | | | 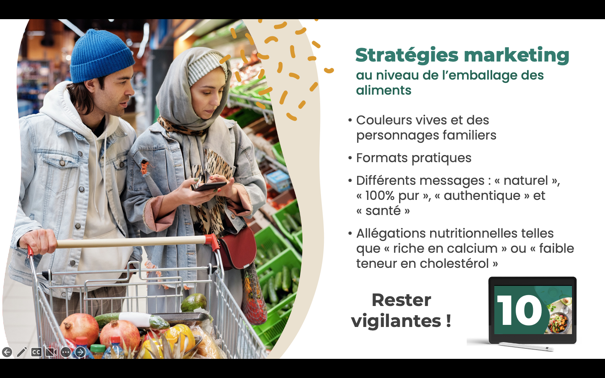 | |
|  |  |  | 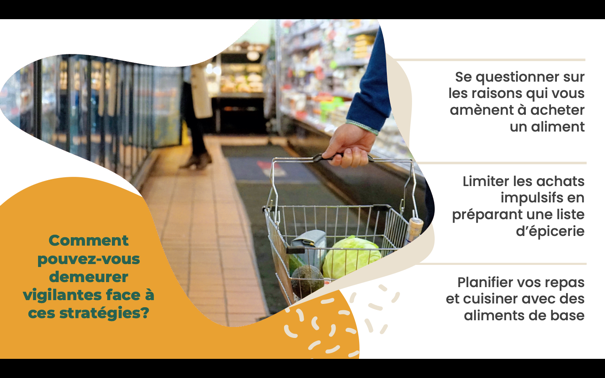 | | | | | | | | 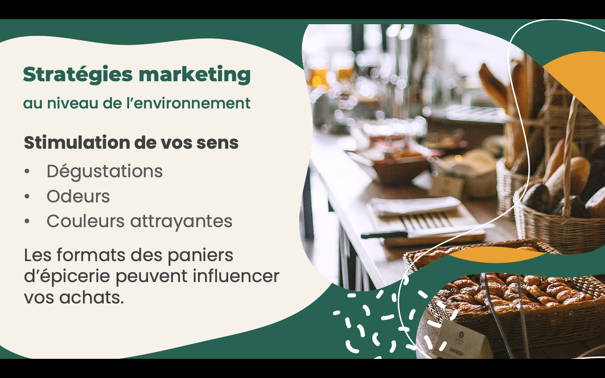 | |
| References | | | 1. Gouvernement du Canada (2021) *Guide alimentaire canadien* <https://guide-alimentaire.canada.ca/fr/>  2. Fondation OLO (2018) *Mieux comprendre le marketing alimentaire pour faire de bons choix à l’épicerie.* <https://fondationolo.ca/blogue/alimentation/mieux-comprendre-le-marketing-alimentaire/> | | | | | | | | | |
| **Video clip 8 - OPTIONAL** | | | | | | | | | | | | |
| Theme | Discomforts during pregnancy: what is the role of nutrition? | | | | | | | | | | | |
| Duration | 10:32 | | | | | | | | | | | |
| Aims | 1. To present dietary strategies to reduce the discomfort of pregnancy symptoms. | | | | | | | | | | | |
| Content | - Natural and normal physical changes throughout pregnancy can cause some discomfort. Even though they are usually harmless, some can be difficult to bear (1,2). - As stated in ***Video clip 1***, healthy eating habits during your pregnancy are good for you and your baby. Some of the discomforts you experience during pregnancy can be helped by diet (3,4). - Appetite, food cravings and aversions (2):   - “Appetite can vary from woman to woman, from pregnancy to pregnancy and even from day to day. The physical changes you experience can have an impact on your appetite. In early pregnancy, hormonal changes can increase your appetite, even if your needs are not greater. As pregnancy progresses, the uterus compresses your stomach and your digestion is slowed, which can decrease your appetite” (2).   - Cravings: Most pregnant women experience cravings during pregnancy, which is a strong urge to eat a particular food. Sometimes these cravings are for chocolate, salty snacks, ice cream or candy. Sometimes it's more nutritious foods, like fruit or dairy products (2).     - Tips: Eating regularly can help you reduce the frequency of cravings. However, no matter what type of craving you have, the important thing is to eat well in general (2).   - Aversions: Where you may find that a food you used to like is less appealing during pregnancy (2).   - Dislikes are most common during the first trimester of pregnancy (2).     - Tips: If you have removed foods from your diet in the first trimester, you can always try them again later in your pregnancy (2).     - If cravings or aversions are very frequent, complicate your diet or make you feel uncomfortable, don't hesitate to talk to your pregnancy professional or consult a nutritionist (2). - Nausea and vomiting:   - Nausea and vomiting (morning sickness) are very common during pregnancy (from 50 to 70% of pregnant women). Although nausea is worse in the morning for some women, symptoms can occur at any time. Most women usually start to feel better after their first trimester, but for others, the nausea continues throughout the pregnancy (1,2).   - Nutritional tips:     - If possible, eat lightly before you get up in the morning (i.e. crackers or toasts) and get out of bed slowly. If not, eat early after getting up (2).     - Limit large meals. Eat more often but in smaller quantities. Avoid going without food for a long time (1,2,3).     - Eat foods that you enjoy and can tolerate easily (1,2).     - Avoid strong odors and foods that bother you (2,3).     - Limit fried or fatty foods (1).     - Eat cold and/or liquid foods (1,2).     - Drink between meals and snacks rather than during meals (1,2).     - Avoid lying down after meals, wait 1 to 2 hours (3).   - Warning: Consult your healthcare provider if: Nausea or vomiting interferes with your activities; you lose weight, you have signs of dehydration (feeling thirsty, dry mouth, lips, or the nose; less urine than usual; dark urine; dizziness and weakness), you experience severe and persistent vomiting (2,3).   - Heartburn and reflux:   - Heartburn is common during pregnancy because hormonal changes slow down the digestive system. The pressure of the growing baby on your stomach can cause reflux of fluid from the stomach up to your throat (1,2).   - Nutritional tips:     - Limit large meals. Eat more often but in smaller quantities (1,2).     - Eat slowly and chew your food well (1,3).     - Eat protein rich food at every meal (2).     - Avoid spicy, fried, fatty or acid foods (i.e. tomato, citrus), coffee, alcohol and tobacco (1,2,3).     - Drink between meals rather than at meal time (2,3).     - Avoid lying down right after eating and avoid eating or drinking before bedtime. You can also sleep with your head and shoulders elevated (1,2,3). - Bloating:   - The hormonal changes that occur during pregnancy slow down digestion, giving bacteria more time to produce gas (1).   - Nutritional tips:     - Limit large meals. Eat more often but in smaller quantities (1,2).     - Eat slowly and chew your food well (1,2).     - Limit gas-producing foods (i.e. beans, cabbage, broccoli, legumes, turnips, peppers, garlic, onions, leeks, certain fruits, etc) (1,2).     - Prefer cooked vegetables and fruits to raw ones (2).     - Avoid chewing gum and soft drinks (1,2).     - Exercise regularly (i.e. after eating, opt for a short 10-15 minute walk if your condition allows) (1,2). - Constipation:   - Constipation is common (40 % of pregnant women) during pregnancy, especially in the second and third trimesters, because food moves through your body more slowly during this time (1,2).   - Nutritional tips:     - Gradually increase your intake of high-fiber foods such as vegetables, fruits, whole grains, beans, lentils, nuts and seeds (1,2). *Refer to* ***Video clip 9****.*     - Drink plenty of water (i.e. at least 2 L/day) (1,2,3).     - Exercise regularly, adapted to your condition, to stimulate transit (1,2,3).     - Go to the bathroom whenever you need it (2). - Fatigue and insomnia:   The hormonal changes and discomforts can make it difficult to sleep (1,3).Most women experience fatigue during pregnancy, especially during the first and third trimesters. During the first few months, your body is supporting the rapid growth of your baby and undergoing many hormonal changes. (1,2). The hormonal changes and discomfort of pregnancy can make it difficult to sleep. (1,3)   - - Nutritional tips:     - Eat a balanced diet (1,2). *Refer to* ***Video clips 2,3,6.***     - Drink plenty of water (1,2).     - Avoid stimulating foods and beverages (coffee, tea, chocolate, cola-type soft drinks) (3).     - Avoid eating fatty foods in the evening (3).     - Eat a full meal at dinner and a light, nutritious snack before bed to avoid hunger at night (3). | | | | | | | | | | | |
| Visuals | 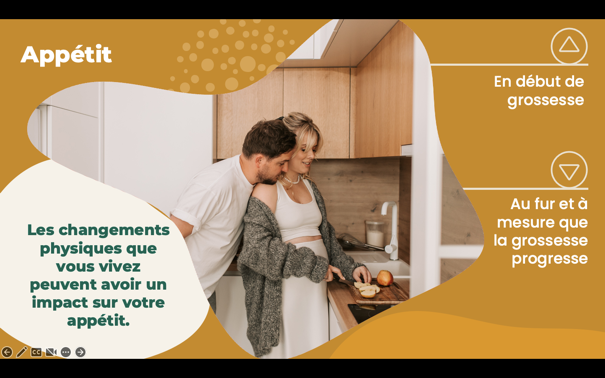 | | | | | | | 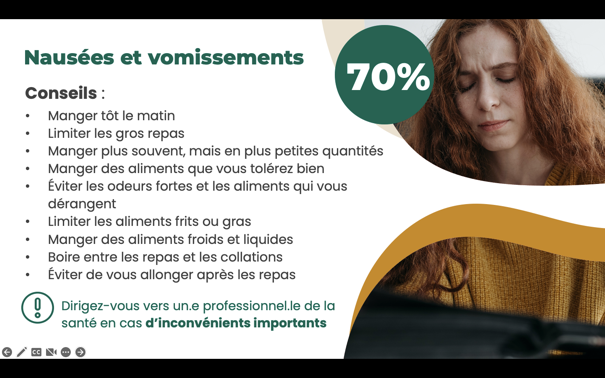 | | | | |
|  | 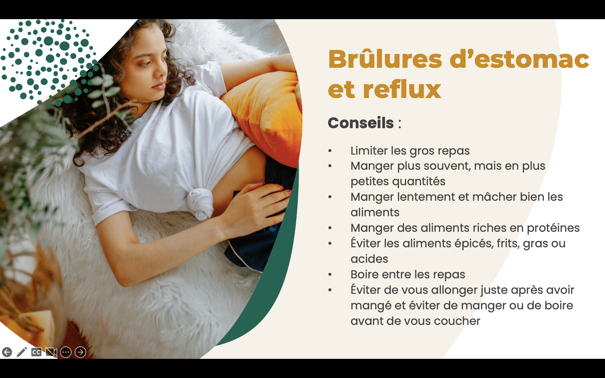 | | | | | | | 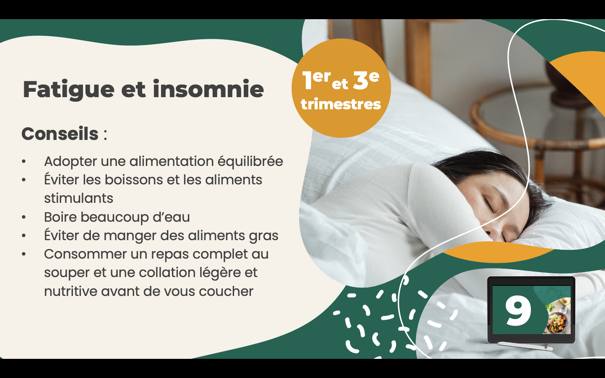 | | | | |
| References | 1. Gouvernement du Canada. (2021) *Votre guide pour une grossesse en santé.* Agence de la santé publique du Canada. <https://www.canada.ca/fr/sante-publique/services/promotion-sante/grossesse-sante/guide-grossesse-sante.html>  2. Doré, N et Le Hénaff, D. (2020) *Mieux vivre avec notre enfant de la grossesse à deux ans : guide pratique pour les parents.* Québec, Institut national de santé publique du Québec.  3. Fondation OLO (2018) *Astuces pour soulager les maux de grossesse.* https://fondationolo.ca/blogue/alimentation/bien-manger/astuces-soulager-maux-de-grossesse/  4. Fondation OLO (2018) *Je suis enceinte : quoi faire?* <https://fondationolo.ca/blogue/alimentation/bien-manger/je-suis-enceinte-quoi-faire/> | | | | | | | | | | | |
| **Video clip 9 - OPTIONAL** | | | | | | | | | | | | |
| Theme | | Nutrients for a healthy diet | | | | | | | | | | |
| Duration | | 10:57 | | | | | | | | | | |
| Aims | | 1. To define and explain the basic roles of the macronutrients. 2. To get participants to balance macronutrients in their diet. | | | | | | | | | | |
| Content | | - Macronutrients are the nutrient components of food that the body needs to provide energy and maintain the body's structure and systems. - **Carbohydrates**: - Functions: Provide you and your cells with energy (i.e. calories) (1,2). - Can be classified in two groups: simple and complex (1,2).   - - Simple: they are made up of small molecules. They usually have a sweet taste (2).     - Complex: they are made up of larger and more complex molecules. They have a non-sweet taste (2).     - Sources:     - Simple: fresh fruits, some vegetables (i.e. carrots), milk, sugar, honey, maple syrup and all sweetened confectionery and beverages.     - Complex: grain products (i.e. pasta, bread, rice), roots (i.e. potatoes), legumes, fruits and vegetables (1,2,3). - Nutritional tips: Choose foods that are rich in complex carbohydrates, which generally contain more fiber and are full of vitamins and minerals (i.e.: whole grain products (rolled oats, brown rice, whole wheat bread, etc.), fruit, vegetables, and legumes (beans, soy, lentils, chickpeas, etc.)) (1,2,3). Since complex carbohydrates are absorbed more slowly than certain simple carbohydrates, the delivery of glucose to your cells will take longer. Result: goodbye energy slumps between meals (1)! - **Fibers**   - - Fiber is part of the carbohydrate family. But, unlike starch or sugar, fiber is a complex sugar that cannot be digested or absorbed by the body (9,10). They therefore arrive intact in the large intestine where they are fermented by intestinal bacteria. The health benefits of fiber are derived from this fermentation (9).     - Functions: Satiety, adequate digestion (prevent constipation), etc.     - Sources: whole grain products, fruits and vegetables, legumes, nuts, seeds (3,9,10).   - Nutritional tips:     - Gradually increase your intake of high-fiber foods and drink plenty of water. This will help you avoid unpleasant digestive problems (9).     - To increase the fiber content of your diet you can:   - Eat washed fruits and vegetables with the skin on (since fibers are found in majority in the skin of fruits and vegetables) (3,10). *Refer to* ***Video clip 4****.*  - Choose plant-based protein more often (lentils, beans, chickpeas, etc.) (6,10).  - Choose whole grains. Swap white rice by whole grain rice, white bread by whole grain bread, and regular pasta by whole grain pasta, etc. (10).  - Create balanced meals where 50% of the food on your plate are vegetables and 25% of the food is whole grains (6). *Refer to* ***Video clip 3****.*  - Favor whole fruits instead of fruit juice (10).   - - - - Substitute part of the white flour in your preparations for semi-complete or complete flour (10). - **Proteins:** - Functions: source of energy for the renewal of muscle tissue, skin, bone tissue, hair and nails, strengthen the immune system (4,5). During pregnancy, proteins contribute, among other things, to the formation of the baby's organs and muscles (3). They provide the material needed for the development of the fetus (4). Protein foods also help maintain energy levels between meals and throughout the day and contribute to the production of breast milk (3,4). - Why is protein so important? Because your body cannot store protein, it needs to get it from food all the time (4). That's why it's important to eat protein at every meal and snack. (3) - Sources:   - - Animal products: Meat, fish, eggs, dairy products.     - Plant-based sources: Legumes, nuts, seeds, grains, enriched soy beverages. - Nutritional tip: Include legumes in your diet more often. They contain less saturated fat than animal foods and decrease your risk of developing cardiovascular disease and cancer (4,6).   - - How to eat more plant-based protein foods (6): Add silken tofu to a soup to make it thicker and creamier; try a bean salad, lentils, rice pilaf or a bowl of vegetarian chili for dinner; make your own trail mix by combining your favorite whole grain cereal with a handful of nuts and seeds; spread hummus on the inside of a whole grain pita and fill it with vegetables like romaine lettuce and shredded carrots; plan a few meatless meals each week. - **Fats:**   - - Despite their bad reputation, fats, also called lipids, are essential to good health (7) and they have a limited role in weight gain when they are of good quality (8).     - Functions: Energy reserve, for insulation and protection of your organs, absorption and transport of fat-soluble vitamins (A, D, E, K), synthesis of hormones and cholesterol, provide essential fatty acids (7).     - Foods contain different types of fatty acids, each with its own qualities and defects. Different types of fatty acids:     - Unsaturated fatty acids (e.g., omega-3s):   - Omega-3 is an essential fatty acid, meaning that it cannot be produced by the body and is necessary for the body's development and functioning (7,8).  - Reiterate that omega-3 fatty acids support the growth of the baby's brain and tissues.  - Specific functions: required for reproduction and growth, formation of cells, integrity of the skin, inflammatory, allergic, vascular, immune reactions, etc., protect against heart diseases (7).  - Sources of Omega-3: cooked low-mercury fatty fish, flaxseed, enriched eggs, canola oil, walnuts (7,8).   - - - Saturated fatty acids:   - Excess amounts increase cardiovascular risk by raising blood cholesterol (7,8).  - Choose foods that have little or no saturated fat (6,8).  - Normally solid at room temperature (7,8).  - Sources: animal products (meat, dairy, butter, lard), palm and coconut oils, hydrogenated margarines, cookies and chips (7,8).   - - - Nutritional tips:     - Remember that not all fats are harmful to your health, but all fats should be eaten in moderation (7).     - Thus, choose foods that contain mostly good fats rather than foods that contain mostly saturated fats such as cooked low-mercury fatty fish, vegetable oils, avocado, nuts and seeds (6,8).     - Solutions to replace saturated fat with good fat: on your toast, replace cream cheese with nut butter; on bread or rolls, replace butter with olive oil and/or balsamic vinegar; to replace spinach or artichoke dip, try making your own hummus or tzatziki; when cooking, replace shortening, lard or hard margarine with healthy oils like canola, olive and soybean (6). | | | | | | | | | | |
| Visuals | | 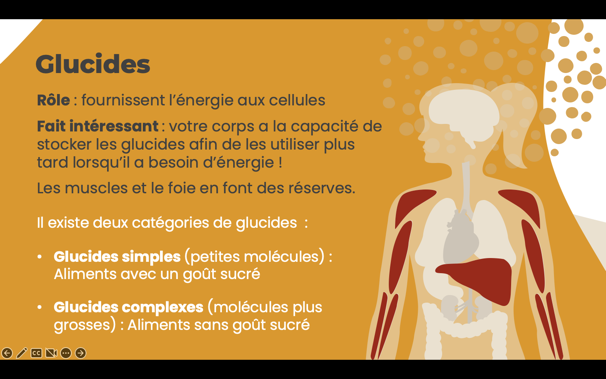 | | | | | | | | 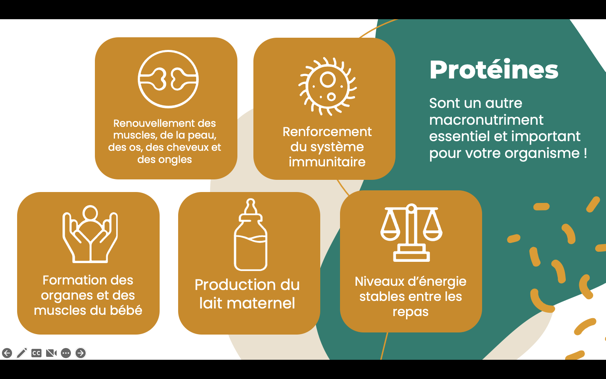 | | |
|  |  | 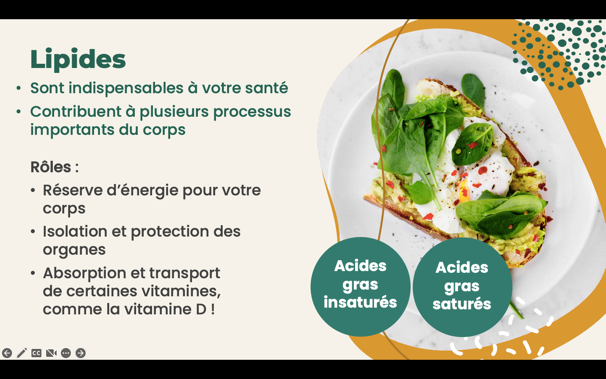 | | | | | | | | 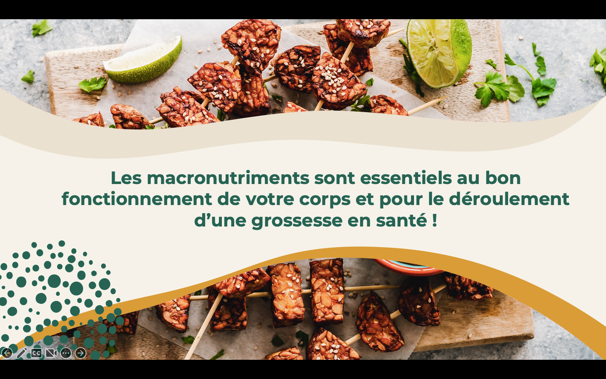 | | |
| References | | 1. Extenso. (2013). *Macronutriments, Glucides*. <https://extenso.org/article/glucides/>  2. Chapon, J. *Les glucides*. Yuka. <https://yuka.io/fondamentaux/les-glucides/>  3. Doré, N et Le Hénaff, D. (2020) *Mieux vivre avec notre enfant de la grossesse à deux ans : guide pratique pour les parents.* Québec, Institut national de santé publique du Québec.  4. Extenso. (2012). *Macronutriments, Protéines*. <https://extenso.org/article/proteines/>  5. Chapon, J. *Les protéines*. Yuka. <https://yuka.io/fondamentaux/les-proteines/>  6. Gouvernement du Canada. (2021) *Guide alimentaire canadien* <https://guide-alimentaire.canada.ca/fr/>  7. Extenso. (2012). *Macronutriments, Lipides*. <https://extenso.org/article/lipides/>  8. Chapon, J. *Les lipides*. Yuka. <https://yuka.io/fondamentaux/les-lipides/>  9. Extenso. (2012). *Macronutriments, Fibres*. <https://extenso.org/article/fibres/>  10. Chapon, J. *Les fibres*. Yuka. <https://yuka.io/fondamentaux/les-fibres/> | | | | | | | | | | |
| **Video clip 10 - OPTIONAL** | | | | | | | | | | | | |
| Theme | How to read nutrition labels to make quality food choices? | | | | | | | | | | | |
| Duration | 9:33 | | | | | | | | | | | |
| Aims | 1. To explain how to read a nutrition label.  2. To get participants to use nutrition labels to make better food choices. | | | | | | | | | | | |
| Content | - Food and nutrition labels are a good way to learn about the nutritional value of foods and can help you make informed choices about healthy and safe foods (1,2). - Food labelling is regulated by Health Canada (1). - You can find good nutrition information on prepackaged foods by looking at their labels, including: nutrition facts table, list of ingredients, nutrition claims, food allergens labelling and shelf life information (1,2). - Benefits of using food labels:   - Food labels provide information you can use to make informed food and beverage choices at the grocery store and at home (2).   - Food labels can thus help you:     - Compare and choose products more easily (to choose products that contain less sodium, sugar or saturated fat) (2,4).     - Know what ingredients and allergens are in a food product (2).     - Choose products that contain a little (low) or a lot (high) of the nutrients you want (2). - Nutrition facts table:   - Provides information about the food for a given serving size, usually the amount consumed in a single meal or snack. This includes the number of calories in that serving and the amount of its major nutrients in grams (g), milligrams (mg), micrograms (µg) or percent daily value (in %DV) (1,2,4).   - The % DV can be used as a guide to show you whether the serving size contains a small (little) or large (a lot) amount of a nutrient (1,2,4).     - 5% DV or less is a little and 15% DV or more is a lot.   - The Nutrition Facts tables allows you to:     - Easily compare two similar products (by comparing the %DV of two different food products with the same serving size, you can choose the healthier one) (1,4).     - Find out how many nutrients and calories a food contains (1,4).     - Identify foods that contain little or a lot of a nutrient (1,4). - Ingredient list:   - Lists all the ingredients in a food product in descending order of weight. The ingredient with the largest amount is listed at the beginning of the list, while the ingredient with the smallest amount is listed at the end (1,2,4).   - Major allergens, gluten sources and sulfates are listed in the ingredient list of most packaged foods. They generally appear at the end of the list (2).   - It is both important and useful to read the list of ingredients. It can help you to: (4)     - Check if a food product contains a particular ingredient.     - Avoid certain ingredients if you have a food allergy or intolerance.   - Note that some nutrients, such as saturated and trans fats, sodium and sugar, may appear under different names in the ingredient list. i.e. the terms corn syrup, dextrose, evaporated cane juice, fructose, invert sugar and maltose are used to name sugar (other examples will be given) (4). - Nutrition claims:   - A message that appears on a food package. Manufacturers generally use two types of messages or claims:     - Nutrient content claims: to describe the nutritional value or benefit of a food. For example, "*Good source of iron*" or “*low in cholesterol*” on the package (1).     - Health claims: to describe the health benefits of a food or certain types of foods. For example, "A healthy diet with a variety of vegetables and fruit may help reduce the risk of certain types of cancer" on the package (1).   - Claims are subject to certain rules set out by Health Canada. All foods carrying a claim must meet certain criteria, but some products may not carry a claim even if they meet the criteria since the presence of claims on food labels is optional (1,2).   - Other claims have appeared in recent years on the packaging and price labels of foods sold in supermarkets. These claims are not developed by the government. They take the form of symbols or logos, or words such as "Good for you" or "Healthy choice" (1).   - How to use claims to make healthier food choices?     - A nutrient content claim can be helpful in choosing foods that contain a nutrient you want to reduce or increase your intake of. For example, look for phrases such as: *free* (for sodium and trans fat) *very high, excellent, high, or good source* (for protein, omega-3, iron) (4).     - A health claim can help you choose foods that you want to include in a healthy diet to reduce the risk of chronic disease. Here's an example: A healthy diet with a wide variety of vegetables and fruit may help reduce the risk of some types of cancer (4). - Warning: claims on food packaging can give you relevant information about the product but be careful. Don't rely on claims alone to make informed food choices (1). Keep in mind that because health claims are optional and only target a few key nutrients or foods, you should still refer to the nutrition facts table to make the decisions that are best for you (4). | | | | | | | | | | | |
| Visuals | 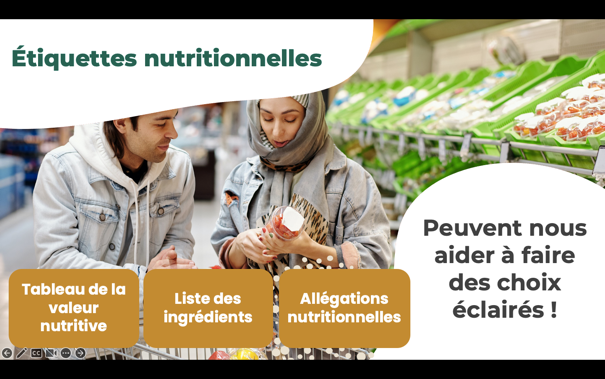 | | | | | | | | 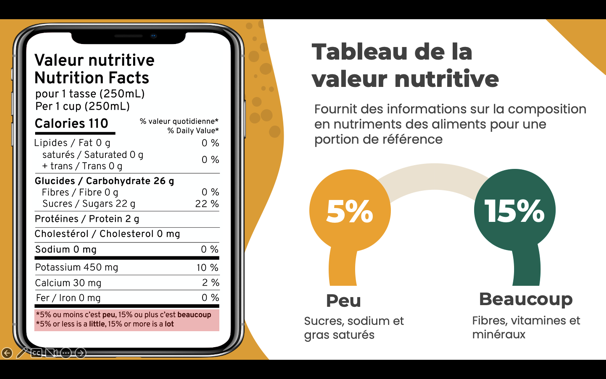 | | | |
|  | 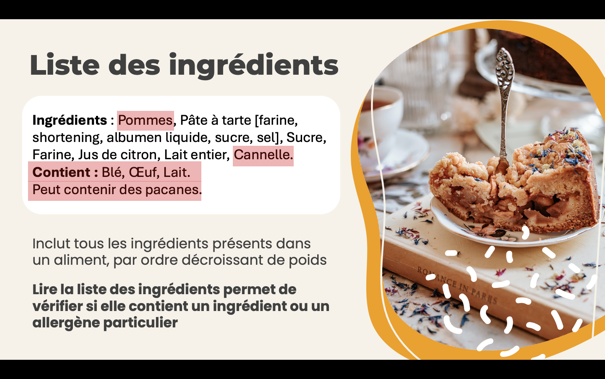 | | | | | | | | 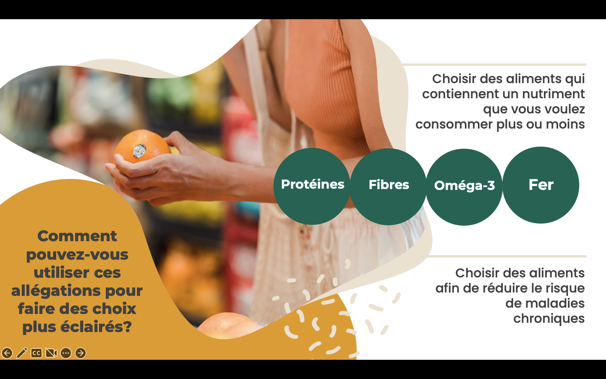 | | | |
| References | 1. Gouvernement du Québec (2019) *Comprendre les étiquettes des aliments.* <https://www.quebec.ca/sante/alimentation/saines-habitudes-alimentaires/comprendre-les-etiquettes-des-aliments>  2. Gouvernement du Canada (2021) *Guide alimentaire canadien* <https://guide-alimentaire.canada.ca/fr/>  3. Gouvernement du Canada. (2021) *Guide alimentaire canadien, Une saine alimentation pendant la grossesse et l’allaitement.* <https://guide-alimentaire.canada.ca/fr/conseils-pour-alimtation-saine/grossesse-allaitement/>  4. Gouvernement du Canada (2021) *Comprendre l’étiquetage des aliments.* <https://www.canada.ca/fr/sante-canada/services/comprendre-etiquetage-aliments.html>  5. Gouvernement du Canada (2019) *Durée de conservation sur l'étiquette des aliments préemballés*, Agence canadienne d’inspection des aliments.  <https://inspection.canada.ca/exigences-en-matiere-d-etiquetage-des-aliments/etiquetage/consommateurs/duree-de-conservation/fra/1332357469487/1332357545633> | | | | | | | | | | | |
